# The “Water Problem”(*sic*), the Illusory Pond and Life’s Submarine Emergence—A Review

**DOI:** 10.3390/life11050429

**Published:** 2021-05-10

**Authors:** Michael J. Russell

**Affiliations:** Dipartimento di Chimica, Università degli Studi di Torino, via P. Giuria 7, 10125 Turin, Italy; michaeljrussell80@gmail.com

**Keywords:** peptide membrane, fougerite/green rust, Hadean Ocean, hydrazine, mushy mantle, submarine alkaline vents, emergence of life

## Abstract

The assumption that there was a “water problem” at the emergence of life—that the Hadean Ocean was simply too wet and salty for life to have emerged in it—is here subjected to geological and experimental reality checks. The “warm little pond” that would take the place of the submarine alkaline vent theory (AVT), as recently extolled in the journal Nature, flies in the face of decades of geological, microbiological and evolutionary research and reasoning. To the present author, the evidence refuting the warm little pond scheme is overwhelming given the facts that (i) the early Earth was a water world, (ii) its all-enveloping ocean was never less than 4 km deep, (iii) there were no figurative “Icelands” or “Hawaiis”, nor even an “Ontong Java” then because (iv) the solidifying magma ocean beneath was still too mushy to support such salient loadings on the oceanic crust. In place of the supposed warm little pond, we offer a well-protected mineral mound precipitated at a submarine alkaline vent as life’s womb: in place of lipid membranes, we suggest peptides; we replace poisonous cyanide with ammonium and hydrazine; instead of deleterious radiation we have the appropriate life-giving redox and pH disequilibria; and in place of messy chemistry we offer the potential for life’s emergence from the simplest of geochemically available molecules and ions focused at a submarine alkaline vent in the Hadean—specifically within the nano-confined flexible and redox active interlayer walls of the mixed-valent double layer oxyhydroxide mineral, fougerite/green rust comprising much of that mound.

*Central to understanding “living mater” is appreciating its sheer improbability. [1].*

*It is through functional properties, not structure, that the organization of a purposive system is expressed. [2].*

## 1. Introduction

The recently revived case for a prebiotic soup in a wet-dry “warm little pond” as life’s womb is—according to a recent article in Nature [3]—driven by “scepticism about Russell’s alkaline-vent hypothesis” as it supposedly “lacks experimental support” and moreover, that the “evidence doesn’t exist” [3]. “By contrast, chemical experiments that simulate surface conditions have made the building blocks of nucleic acids, proteins and lipids” [3]. Further, the warm little pond “offers a solution to a long-recognized paradox: that although water is essential for life, it is also destructive to life’s core components” [3]. The further charges variously stated are (1) that prebiotic “molecules wouldn’t survive long in those (alkaline vent) conditions”, (2) that “the formation of these protopeptides is not very compatible with hydrothermal vents” [3], and (3) “None of that synthesis exists in that deep-sea hydrothermal vent hypothesis. It just simply hasn’t been done, and possibly because it can’t be done,” says Catling [3] and, (4) on top of it all Sutherland opines “You can say with some degree of confidence we need to be on the surface, we can’t be deep in the ocean or 10 kilometres down in the crust” … “Then we need phosphate, we need iron. A lot of those things are very easily delivered by iron–nickel meteorites” and “once RNA, proteins and so forth had formed, evolution would have taken over and enabled proto-organisms to find new ways to make these molecules and thus sustain themselves” [4]. 

In 2017, Sutherland officiated at the submarine AVT requiem in a Nature Reviews, Chemistry paper declaring; “A requirement for ultraviolet irradiation to generate hydrated electrons would rule out deep sea environments. This, along with strong bioenergetic and structural arguments, suggests that the idea that life originated at vents should, like the vents themselves, remain ‘In the deep bosom of the ocean buried’” [4]. It appears that to make room in the trend-setting journals for what was assumed by many to be a dead duck, required the peremptory demise of AVT. For example, from these imputations we read “experimental support is growing for the idea that life started in small bodies of water on land” [3]. Furthermore, the case for the “pond” has even earned Catling’s blessing: “There’s a lot of work that’s been done in the last 15 years which would support … ‘surface lakes and puddles’… which are highly promising …” [3]. 

Are they? Here we first argue that the boot is on the other foot; that an ultraviolet UV-energized, wet-dry cycling pond—the alternative Hadean open-womb proposed for life’s “origin”—is a reductionist fantasy dreamt up in the absence of geological and thermodynamic consideration. Hence, as this self-referencing pond argument makes its parochial rounds it never finds a home in the biological literature. We will call a variety of witnesses to speak against this “origin of life” pretender and further caution the little pondists of the statistical understanding of thermodynamics, the second law included, known since Boltzmann revealed it to us all back in the eighteen seventies, is fundamental to all dissipative structures in the Universe, and that ignorance of this law is no excuse for endlessly propounding ‘origins scenarios that flatly violate it’ [5]. We then expose this “false requiem” being played for the submarine alkaline vent theory for life’s emergence for what it is, before playing our own overture to emergent life.

## 2. Evaporating Pond Theory of Life’s “Origin”

David Deamer has long championed subaerial volcanic hot springs exhaling into shallow ponds subject to evaporation as the birthplace of life. “These wet-dry cycles are everywhere,” says Deamer … “It’s as simple as rainwater evaporating on wet rocks” [3]. “Deamer has conducted several experiments in modern volcanic hot springs to test his ideas. In 2018, his team showed that vesicles could form in hot spring water and even enclose nucleic acids, which Marshall reports “would not form in seawater” [3,6]. “Previously, Deamer and his team in 2008 [7] had mixed nucleotides and lipids with water, then put them through wet-dry cycles. When the lipids formed layers, the nucleotides linked up into RNA-like chains—a reaction that would not happen in water unaided. A follow-up study found that when the resulting vesicles were dried, nucleotides linked up to form RNA-like strands [8]”. They conclude, “wet-dry cycles on the edges of the pools would have driven the formation and copying of nucleic acids such as RNA [8]”. An “alternate chemiosmotic energy” develops in these supposed conditions though, in contrast to AVT as well as life itself, the gradient is the reverse of the prototypical proton motive force! The disequilibria in their model is provided by reduced sulfur compounds inside the lipid vesicles, the electrons from which are then transported through the membrane by the diffusion of quinone carriers “present in the Murchison meteorite” as they make their way to the ferricyanide acceptor on the outside [6]. As mentioned, protons are also “released in the process, producing an acidic interior and substantial gradients over 2 pH units” [9]. 

Like Deamer, Frenkel-Pinter and collaborators [10] argue that wet–dry cycles were crucial. “Dry conditions, she says, provided an opportunity for chain molecules such as proteins and RNA to form. But simply making RNA and other molecules is not life. A self-sustaining, dynamic system has to form.” Frenkel-Pinter suggests that water’s destructiveness could have helped to drive such a system. “Just as prey animals evolved to run faster or secrete toxins to survive predators, the first biological molecules might have evolved to cope with water’s chemical attacks—and even to harness its reactivity for good”. Moreover, the open ocean is unviable, says Frenkel-Pinter, because there is no way for chemicals to become concentrated. “That’s really a problem,” agrees Bonfio. The idea is that, with each cycle of wetting, the weaker molecules, or those that could not protect themselves by binding to others, were destroyed. Bonfio and her team demonstrated this in a study this year, in which they attempted to convert simple fatty acids into more-complex lipids resembling those found in modern cell membranes [11]. The researchers created a mixtures of lipids, and found that the simple ones were destroyed by water, while the larger, more complex ones accumulated.” “At some point, you would have enough of these lipids for them to form membranes,” she says. In other words, “there might be a Goldilocks amount of water: not so much that biological molecules are destroyed too quickly, but not so little that nothing changes” [3].

Although wet-dry cycling has been around since at least the nineteen seventies as a proposed prebiotic mechanism for polymerizing amino acids [9,12,13,14], a similar struggle but with RNA led to the re-opening of Darwin’s casket for the resurrection of “pond theory” [15,16,17,18,19,20,21,22,23,24,25,26,27,28,29,30,31,32]. However, that casket was empty! In a carefully considered footnote to his Origin of Species of 1872, Darwin wrote ‘’It is no valid objection (to the theory of natural selection) that science as yet throws no light on the far higher problem of the essence or the origin of life” [33,34]. 

Nevertheless the literature favoring wet–dry cycles is burgeoning. One example is the “polymer fusion model” proposed by Hud and his collaborators [35]. Appealing to retrodiction from the extant RNA molecule—“the penultimate member of a continuous series of polymers”—they suggest that its ultimate precursor was made from primeval prebiotic “hypothetical pre-RNAs”. These “plausible” entities were assumed to exist in the Hadean and were put together from the organic building blocks of life supplied by chondritic meteorites or Miller–Urey prebiotic reactions in a “drying pool” or “drying lagoon”. “My grandfather’s axe” is the pedagogic metaphor called upon to indicate how the ready-made primeval prebiotic “hypothetical pre-RNAs” assumed to exist in the Hadean composed of (1) recognition units (bases), (2) trifunctional connectors (ribose) and (3) an ionized linker (glyoxylate)—the true and pure forebears of RNA and DNA—came to be [35]. But the metaphor, like the scientific assumption contained in the body of their paper, falls short in just invoking the immediate past, for our *palaeo*-grandfather’s axes—unlike grandad’s—were hand-held and made of flint! In opposition we reiterate that the only way to produce biotic monomers and polymers is to start with the simplest of molecules or ions indisputably present on the early Earth, viz., CO_2_, H_2_O, CH_4_, HPO_4_^3−^, N_2_, NO, NH_3_, Fe, Ni, Co, Zn, Mn, Mg, Mo, Na, K and reduced sulfur [36,37,38,39]. 

Top-down attempts to mask or muzzle AVT have now been joined by van Kranendonk and colleagues’ [31,32] “bottom up” presentations of geological evidence for AVT’s supposed ‘passing’. The “water problem” is front and center of their argument—a prejudice that harks back to Shapiro’s 1986 isolating assertion: “The enemy is water”! [40]. The van Kranendonk preferred scenario is for a site which has the advantage of “wet–dry cycling and greater chemical complexity (achieved through additional air/volcanic gas-rock, and air/volcanic gas-water interactions, and information exchange between the numerous, chemically variable pools that typify hot spring systems), in addition to the acidic conditions required to form lipid membranes” [31]. 

To gather support for their preferred model, van Kranendonk et al. [31] explore “deep-time” in search of amenable conditions for their “origin of life”. It so happened that a 3.5 billion-year-old, anoxic hot spring setting from the Pilbara Craton (Australia)” revealed “that its hydrothermal veins and compositionally varied pools and springs concentrated all of the essential elements required for prebiotic chemistry (including B, Zn, Mn, and K, in addition to C, H, N, O, P, and S)” [31]. Their argument espouses “temporal variability (seasonal to decadal), together with the known propensity of hot springs for wet–dry cycling and information exchange” and suggests that this “would lead to innovation pools with peaks of fitness for developing molecules” [31]. But this geological scenario falls short of life’s likely onset by nearly a billion years and is no more relevant than other such sites! 

## 3. Dirty Chemistry 

The struggles to satisfy the RNA world hypothesis in the pond brought about the concept of “dirty chemistry”. This requires a myriad of organic molecules derived from the Earth, atmosphere or extra-terrestrially, to self-organize themselves somehow into the metabolic cycles, thus supposedly explaining how ‘wonderful life’ originated [28,41,42,43,44]. Ignored were the earlier entreaties of Schrödinger and Prigogine to understand that, as history itself painfully teaches us, order can only be derived from order, or fluctuations therein [45,46,47]. Nor is this what we might call “a hypothesis of least astonishment”, i.e., “neither more, nor more onerous causes are to be assumed than are necessary to account for the phenomena” ([48], p. 482). 

Dirty, or messy chemistry advocates generally call on lipids as the first requirement to establish a cell. For example, Deamer and Barchfeld studied “how lipids, another class of long-chain molecule, self-organize to form the membranes that surround cells” [3,49]. Deamer and Barchfeld [49] “first made vesicles: spherical blobs with a watery core surrounded by two lipid layers … (T)hen … dried the vesicles, and the lipids reorganized into a multi-layered structure like a stack of pancakes. Strands of DNA, previously floating in the water, became trapped between the layers. When the researchers added water again, the vesicles reformed—with DNA inside them. This was a step towards a simple cell” [3,49]. Interesting physics and chemistry? Maybe, but it again avoids the problem of DNA’s ultimate source! 

Other experiments said to support this heterotrophic origin of life have been made or argued for by Rajamani et al. [7], Monnard et al. [50], Deamer et al. [51], Mulkidjanian [52,53], Deamer and Weber [54], Hazen and Sverjensky [55], Kim et al. [56], De Guzman et al. [57], Forsythe et al. [58], Hazen [59] and Pearce et al. [22]. In a similar vein, Powner and collaborators, [16] argue that at “some stage in the origin of life, an informational polymer must have arisen by purely chemical means”. Their publication was particularly impactful, having concluded: “findings suggest that the prebiotic synthesis of activated pyrimidine nucleotides should be viewed as predisposed” (*sic*). In order to demonstrate the verity of this statement, they produced such pyrimidine nucleotides using what they deemed to be “plausible prebiotic feedstock molecules”, viz., cyanamide, cyanoacetylene, glycolaldehyde, glyceraldehyde and orthophosphate. These authors backup the statement with the remark that “the conditions of the synthesis are consistent with potential early-Earth geochemical models.” From the cyanamide, cyanoacetylene, glycolaldehyde, glyceraldehyde and orthophosphate they went on to generate arabinose amino-oxazoline and anhydronucleoside, supposed waystations to the pyrimidine ribonucleotides needed for RNA synthesis! They further remark, “for prebiotic reaction sequences, our results highlight the importance of working with mixed chemical systems in which reactants for a particular reaction step can also control other steps. Although inorganic phosphate is only incorporated into the nucleotides at a late stage of the sequence, its presence from the start is essential as it controls three reactions in the earlier stages by acting as a general acid/base catalyst, a nucleophilic catalyst, a pH buffer and a chemical buffer” on the way to generating “two of the four nucleotides that comprise RNA, starting only with highly concentrated aqueous solutions of phosphate and four simple carbon-based chemicals and cyanamide” [16]. Crucial steps required UV radiation. Thus, they conclude that such reactions could not take place deep in an ocean—only in a small pool or stream exposed to sunlight, where chemicals could be concentrated” [16]. 

What reactions could these be we ask? Well, UV radiation is normally brought to bear as “a photolysis mechanism that favors selection of the most UV-resistant biopolymers: (e.g., oligonucleotide-like polymers at the expense of the bases) [16] though quite how such substrates are produced in the necessarily exponentially rising concentrations that would be required for such an “origin” of life is not considered. UV has also been heralded as an energy source to free-up an electron from tricyanocuprate Cu(CN)_3_ though again, exactly how the latter is produced in sufficient quantities, or at all, is also not demonstrated [60]. The Earth’s volatisphere was simply too oxidized to support a substantial source of that poison [61,62,63].

In spite of this knowledge, Damer and Deamer [9] suggest that gels might make a good ambient environment for such chemistry requiring, as they do, low water activities. They further report that Sutherland’s team [64] “has since shown that the same starter chemicals, if they are treated subtly differently, can also produce precursors to happen in water unaided. Other studies are pointing to different proteins and lipids”. They also appeal to cyanide salts—those well-known poisons (pity the poor ferredoxins) where these reactions might have taken place if water containing these salts was “dried out by the Sun, leaving a layer of dry, cyanide-related chemicals that was then heated by, say, geothermal activity”. We wait with bated breath to hear of similar ‘order-from-disorder’ activated chemistry, perhaps to be revealed by NASA/JPL’s Perseverance on Mars? But to us all this is wishful thinking dreamed up in an RNA penthouse without solid foundations and no visible means of support in the moderately oxidized and completely flooded surface of the Hadean world. Nevertheless, as this speculation has such a grip on the “origin-of-life” community we give it time of day next.

## 4. RNA-DNAology

Sutherland’s team, using energy from sunlight, has recently produced the building blocks of DNA from high concentrations of photoactivated cyanide (HCN), cyanoacetylene (CH_3_N) and hydrogen sulfide (H_2_S), “something previously thought implausible” [3,65]. Again, no geological evidence for such a soup has ever been mustered. Other suggestions along the same lines call upon prebiotically plausible acrolein and 2-aminooxazol which furnish ribo-3-5P with excellent ribo-selectivity through a combination of kinetic and thermodynamic control. 

In a commentary (https://chemistrycommunity.nature.com › posts › 3720, accessed on 25 March 2021) on their paper Bonfio and Mansy [66] also inductively conclude that, in the supposed absence (*sic*) of a “historical record”, the early Earth’s store of electrons required for life resided not in the minerals comprising the Earth *per se* but in a “chemical deposit of NADH” (nicotinamide adenine dinucleotide, C_21_H_27_N_7_O_14_P_2_) (*sic*)! Bonfio and Mansy [66] also relieve origin-of-life theory from the requirement of the early production of the rather complex FMN (flavin mono-nucleotide, C_17_H_21_N_4_O_9_P), suggesting that it too was present in the pond or pool. However, they remarked modestly that they could not be “satisfied” with their model until they had demonstrated that ubiquinone, CoQ_10_ (C_59_H_90_O_4_)—a highly hydrophobic molecule—could dissolve in the lipid membrane: which it duly did! This was exciting for Bonfio and Mansy [66] because in biology too, “the electrons donated from NADH to iron-sulfur peptides” are “further transferred to ubiquinone, which is somewhat similar to how electrons pass from NADH to Complex I and then to ubiquinone.” Moreover, Bonfio and Mansy [66] suggest that the said electrons were drawn to an even better oxidant than the absent oxygen, viz. hydrogen peroxide (H_2_O_2_), which, they say “makes sense”. 

Added to these fortuitous circumstances, according to these same authors, NADH was not the only nucleotide present at that time, because, as it is “generally accepted that RNA and nucleotides were crucial for the origins of life”, there must have been a store of them too! They conclude, “the use of electron carriers that fit within the RNA world hypothesis (is) attractive”. Bonfio et al. [67] also suggest that UV radiation drives the synthesis of iron-sulfur clusters which are crucial to many proteins. We are told these iron-sulfur clusters would break apart if they were exposed to water, but they were found to be more stable if the clusters were surrounded by simple peptides 3–12 amino acids long. Furthermore, peptides were also apparently to be had, though their source is less clear. However, apparently there was an abundance of lipids available, or generated to order—all of the same length and chirality to provide the first bag for the first cytoplasm! Added to this Bonfio and Mansy [66] opine; “iron-sulfur peptides either engaged in reactions that immediately generated a pH gradient or dumped these electrons on intermediate polyaromatic electron carriers (which) would have allowed for a simple proto-metabolism to form in a way that was open to further development.” This is the “messy chemistry” idea writ large, and we reiterate our complaint that a plausible prebiotic source of these so-called activated highly-reduced monomers, some of them extremely complex, has not been established and, to our minds, never existed on the early Earth. Moreover, the always present issue of waste disposal—the ‘entropic pull’—is not addressed.

## 5. AVT Critiques: The False Requiem

Here we counterface all the arguments made in recent papers from the very well-funded and promoted groups militantly opposed to AVT [3,68]. One of these papers offers the advice “Don’t try to prove an idea is right. Instead, try to falsify it” [69]. Fully cognizant of Popper’s “Reason and Refutation” [70], this has long been our own mantra, though notably unshared across the community. As an example of good faith, Branscomb and colleagues [71] wrote, “arguably the key virtue of the alkaline hydrothermal vent (AHV) model as a scientific hypothesis regarding the initial steps in the emergence of life is its essentially unique vulnerability to disproof. It places all of its chips on the claim that certain naturally arising, but experimentally reproducible, geochemical circumstances do produce castles of mineral ‘cells’ in which three key, undeniably life-like chemical disequilibria are ‘abiotically’ generated and maintained. If it proves not to be possible to experimentally substantiate these conjectures, then we may expect interest in the theory to wane.” Furthermore, falsifiable predictions of AVT were listed in Russell [72] that would, if demonstrated, “reveal embarrassing missing links, or even leave the AVT as just one more casualty of the general theory of natural rejection.” We look forward to similar commitment and clarity from the wet-dry polymerizing pond people. However, we do admit to being impressed over the one prediction made by this group—viz., Dimitar Sassalov’s promise that Harvard University “will soon have the equivalent of a living thing in the lab at the chemical level”. We will be particularly interested to hear what bearing such an artifact might have on the putative ‘first universal ancestor’, its evolving progeny and the geochemical/geophysical disequilibria responsible for its emergence [68]?

One prejudice held against AVT is owed to the denial of known Hadean conditions by those who would attempt, as mentioned, the resuscitation of Darwin’s off-the-cuff remark in a letter to Joseph Hooker, now known in Tabloid speak as “Darwin’s warm little pond” [73]. In contrast, the AVT is built solidly in acknowledgement of the geological, geochemical and geophysical conditions on the early Earth as assembled by countless scientists. The retrodicted mineralogy of the early Earth cannot be dismissed merely by writing “it is uncertain whether these (minerals) were available on the prebiotic Earth” [74]. To counter this statement, we present in Table 1 all the evidence pertaining to our contention that the rocky surface of the Earth was always submerged in the roiling Hadean Ocean. Indeed, we challenge those that would favor the putrid pond idea to counter all these aspects of the Hadean Earth listed in Table 1—a world so different than today’s that it is wiser to think of it as a different planet.

Foremost amongst Damer and Deamer’s [9] various objections to submarine alkaline hot springs also depends on that so-called “water problem”. For example, they argue that, “as a general rule, the much higher concentrations of ionic solutes composing seawater inhibit self-assembly of membranous structures and encapsulation of polymers.” Furthermore that, “the water activity within a submerged mineral cavity … will be at equilibrium with the surrounding ocean bulk.” “This presents a significant thermodynamic hurdle because in aqueous solutions condensation reactions leading to polymer synthesis would require chemically activated monomers such as the nucleoside triphosphates that drive biological metabolism or the imidazole esters of mononucleotides used in the laboratory.” They continue, “a plausible prebiotic source of activated monomers has not been established experimentally.” However, as has long been known, there is no reaction-driving free energy in a single phosphate-reactant bond! The free energy is in the displacement from equilibrium of the pyrophosphate/phosphate-ratio. Still Damer and Deamer go on, “due to the aforementioned water problem, should any catasanolytic polymer, let alone one so complex as a primitive ATP synthase, be formed by chance in a vent environment, without the constant repair and resynthesis by the enzymes of biology, it would soon be disassembled by hydrolytic decomposition.” (Given the problem of “constant repair and resynthesis”, we wonder how the process of repair would be managed as life was supposedly ‘birthing” in an evaporating pool?) They add, “cycling of systems of polymers … that can drive molecular evolution along the path to cellular life … is not available in a continuously immersed environment.” Moreover, such “sites are compromised because of the uniform, dilute nature of the ocean reservoir and its limited capacity to concentrate either the simple organic compounds or the trace elements required for prebiotic chemistry” [9].

Damer and Deamer’s complaints are echoed, as we have noted, by Frenkel-Pinter and Bonfio [3], and have also been reiterated by Voosen [180]. Van Kranendonk et al. [31] take up the same cry, calling upon Mulkidjanian et al. [17], Hud et al. [35] Ross and Deamer [20] and Deamer et al. [51] for support when they write “Oceans are also considered unlikely sites for OoL due to their limited capacity for complexity, the high salt and total divalent cation (e.g., Ca^2+^ and Mg^2+^) concentrations that inhibit lipid membrane assembly and protocell formation, and because organic polymer formation requires condensation reactions at sites where wet–dry cycling can take place (‘‘The Water Problem’’).” However, this lipid argument has no phylogenetic support nor any bearing on the submarine alkaline vent theory. 

In AVT, the first membranes comprise, for example, multilayered enantiomeric 16-mer residues such as Ala-Glu-Ala-Glu-Ala-Lys-Ala-Lys amyloid beta-sheet peptides [181]. Moreover, Ser-Gly-Ala-Gly-Lys-Thr-alpha sheets that we also favor are so much more functional, even as information molecules [163,181,182,183,184,185]. These peptides can also act as alpha-helix P-loops [181,186,187,188,189,190,191,192]. Therefore, the Damer–Deamer complaint regarding the supposed problem of encapsulation does not apply to peptide membranes which are known to precipitate in such environments [9,191,192]. Surely, the assertion that condensation reactions (or catalysis in general) cannot take place in water could be read to assume life’s wet cells are also unviable! The nanochemistry and nanotechnology literature appears to have passed these complainants by! In AVT’s defense redox catalysis and polymerization can be promoted within nanometer-sized pores and interlayers such as to be found in layered double hydroxides, including fougerite (~green rust), silica films, amyloid and peptide nests [72,193,194,195,196,197,198,199,200,201,202,203,204,205,206,207,208,209,210,211,212,213,214,215,216]. Moreover, it should be recalled that the AVT is the only theory which proposes a viable and explicit mechanism for the generation of out-of-equilibrium pyrophosphates as we will address in the next section.

Damer and Deamer [9] exclaim that “the experimental evidence and thermodynamic models of the vent hypothesis have recently been challenged and these critiques should (also) be addressed [9]. This seems particularly egregious; the facts are that the Jackson “criticism” [217,218] had already been thoroughly rebutted by Lane [219]; the explanation for how life left the vent environment was previously detailed in Russell and Hall [156,159], and the Ross [220] criticism has been exhaustively excoriated by Branscomb and Russell [221] without rejoinder. Wächtershäuser’s [222] challenges apply to the pond theories more than they do to the AVT and we deal with them in their own right in the next section. But first we can report on their reasonable challenge [9] that “one such test … for the vent scenario is that carbon dioxide can be reduced to simple organic solutes such as formic acid in a vent environment” [223,224]. Such a test has now been experimentally demonstrated both in the serpentinizing system and the vent environment [143,225].

A further directive is that “(H)ypotheses for an origin of life must also propose that a cell-sized compartment is able to maintain sufficient concentrations of reactants so that metabolic reactions can be initiated.” This challenge has also been met both theoretically [226,227], as with recognition that “surface area available for catalytic processes exceeds that of a solid crystal by orders of magnitude”, and in experiments that show the product exceeds that to be expected of the mere surface of green rust/fougerite [227,228,229,230,231,232], products that include ammonium, amino acids and, perhaps, hydrazine [185].

**Figure 1:**
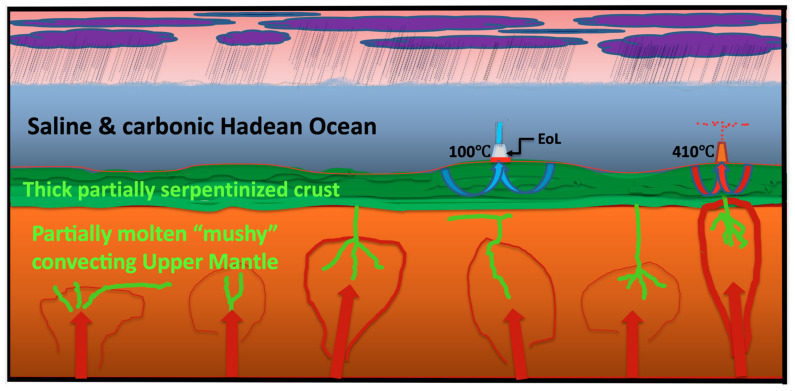
Depiction of our Hadean planet. The crust was completely submerged with a ~5 km deep ocean as the magma ocean was still too mushy to support significant bulges even at the apices of mantle plumes [98,99,100,101,102,106,233,234,235] EoL: emergence of life.

While it is admitted by Damer and Deamer [9] that the Hadean Earth did not have continents, they do argue that it was likely to have volcanoes similar to those from the same era still visible on Mars. Volcanoes yes, tens of thousands of them probably, but given the mushy state of the mantle [98] and ergo its limited load-bearing capacity, the salience of plume-related large igneous provinces and the tumultuous weather of the era, then the idea of Hadean volcanoes hosting fresh water ponds is, in Wächtershäuser’s terminology, a “pre-falsified” theory” one “that falls stillborn off the press” [222] (Table 1, Figure 1). Further, the view that the “concentrating potential” of reactants, e.g., “amphiphilic compounds”, in such a pond adds significant free energy to a system that can be used to drive condensation reactions flies in the face of how entropy is reduced in general [47], let alone how these putative reactants would be produced in the exponentially increasing concentrations required by life’s procreation and evolution!

It seems to have escaped our critics that the AVT is not an “origin” story but a theory of “emergence” of a unique dissipative structure [1,226] because “organismic wholes cannot be built piecemeal from molecular parts, and the “whole provides rules and contexts in which parts emerge and acquire functional significance” [1]. The RNA world’s opposition to the AVT is still argued despite (because of?) the several cogent refutations of the RNA world by and repudiations of soup theory [236,237,238,239,240,241,242,243,244]. These objections to the wet/dry RNA pond models have been comprehensively ignored and remain to be answered. It seems there is more to be had by challenging the counter theory—the AVT—rather than facing up to the proverbial mote in the eye. 

In the Damer–Deamer [9] critique we further read not only that the AVT could not survive the dilution (of organic molecules) that inevitably would occur in a global salty ocean, but also that “seawater is too salty” to let lipids come together to form membranes and threatening the stability of any of those that threaten the stability of lipid membranes. However, to counter this view, Jordan and his coworkers [245] have demonstrated the viability of lipids to do just that, though they do not specify a lipid source. Damer and Deamer [9] carry on with the suggestion that the cycling of systems of polymers through distinctive dry, wet, and moist phases will drive molecular evolution along the path to cellular life, “a process that is not available in a continuously immersed environment”.

It is noteworthy here to emphasize that, notwithstanding the text book diagrams showing lipids to dominate the cell membrane, they barely constitute 20 to 30% of these structures; that role is mainly taken by the proteins. Their remark is also irrelevant to AVT anyway as the lipids in archaea and bacteria have opposite chirality and the split is likely to have been after the last universal common ancestor (LUCA) [246,247,248,249]! Nevertheless, we read that “submarine hydrothermal vents represent a later adaptation for extremophilic microbial life that can thrive in conditions vastly different from the clement pools where life can begin” [9]. This tempts us to ask the same question put by the poet in the biblical book of Job, “Have you descended to the springs of the sea or walked in the unfathomable deep … Have you comprehended the vast expanse of the world?” ([250], p. 192).

## 6. Wächtershäuser’s Probe

In contrast to the rather loose criticisms of the little pond people, Wächtershäuser’s are quite precise [222]. For example, he writes, the “ingenious FeS-membrane theory (Russell et al. 1989 [36]; Russell and Hall 1997 [157]) postulates an open cell structure within a precipitated mound of FeS at the bottom of the primitive ocean” but then charges that the microphotograph used to demonstrate such structure was in reality an artifact of freeze-drying. This is as maybe, but more recent experiments that also consider green rust precipitation, belie this charge [232]. A further criticism, that concerning the supposed instabilities in a hydrothermal mound, is grounded in the assumption that any organic polymers produced there are unstable. Yes, they would be if it weren’t for the fact that water activities would be so low in the nanoconfined spaces in fougerite/green rust and within the subsequent peptide nests as to possibly promote condensation reactions, while there would still be water enough for necessary hydrolyses to proceed in that same environment [72,201,251]. 

With respect to mineral membranes in general [252,253] Wächtershäuser [222] also doubts that they could hold a pH gradient sufficient to drive, for example, phosphate condensation in an approximation of the proton motive force as well as a delta Eh sufficient to drive other protometabolic processes. Our expectation was that orthophosphate driven into green rust interlayers would, as in pyrophosphatase, condense to pyrophosphate in the conditions obtaining at the alkaline vent [72,100,123]. To the former challenge Qingpu Wang and his coworkers [254] have recently demonstrated just such a condensation of ortho- to pyrophosphate in a microfluidic device driven by a delta pH of 3.6. Nevertheless, we readily admit that other biology-like condensations await further experimental testing and demonstration [255]. The remainder of Wächtershäuser’s [222] criticisms make much of Hadean conditions which are more directed to the RNA world proponents and anyway are dealt with in some detail below. However, still missing from Wächtershäuser’s [222] diatribe is a status report on his own “pyrite hypothesis” for the “origin of life” [256].

## 7. The “Pond” in the Hellish Hadean

*Pace*, Sleep and collaborators’ [257] and Damer and Deamer’s [9] opinions, there were no “clement surfaces”, or “clement pools” to be had on the surface of our Hadean planet—that young water world, impacted as it was by high energy UV, X-rays, meteorites and asteroids, was no place to conceive and succor life. On the contrary, that young world was spinning at such a rate—a day likely lasted less than 8 h—and the moon was so close as to engender perpetual hurricanes, endlessly roaring 10 m high storm waves and rapid tidal oscillations in an ocean with twice the present volume [108,109,110,111,112,113,114,115,116,117]. However, we read in Damer and Deamer [9] that volcanoes “emerging through a global ocean would be the original land masses on the Hadean Earth analogous to Hawaii and Iceland” with “abundant hydrothermal fields with multiple hot spring systems replenished by precipitation evaporating from the surrounding ocean. The distilled fresh water would percolate into hot rocks and then circulate back to the surface as springs and geysers. Hydrothermal fields provide sources of heat and chemical energy to drive polymerization reactions in films of concentrated organic solutes that form on mineral surfaces during repeated cycles of wetting and drying.” 

Travelling back to when the Universe was only two thirds its present age we would be observing a very different planet where surface conditions were unrelentingly tumultuous; the likely depth of the Hadean Ocean was about 5 km; and the mushiness of the upper mantle could not support notional ‘Icelands’ or ‘Hawaiis’, with their supposed tidal pools, ponds or land-locked seas as sites for the origin of life [98,99,100,101,102,103,104,105,106,107,108,109,110,111,112,113,114,115,116,117]. Even so Carrell opines that a “larger ocean exacerbates the biggest strike against the underwater scenario: that the ocean itself would have diluted any nascent biomolecules to insignificance.” [180]. No mention is made of the autogenic emergence of life favored by scholars of early metabolism and as assumed in the submarine AVT—that is through the generation of organic molecules from the simplest of carbon-bearing precursors from the bottom up, in the hydrothermal mound precipitated at the alkaline vent [36,38,39,72,166,185,223,224,225,226,227,239,240,247,248].

That the earth’s atmosphere has been mildly oxidized and oxidizing over the last 4.4 Ga is because the redox state of carbon in the quartz-feldspar-magnetite buffered hot upper mantle is as carbonate. This seems surprising given that the Earth is largely an amalgam of metal-bearing chondrites, many of them carbonaceous. The reasoning goes that as the olivine-rich mantle is subjected to pressures beyond ~21 GPa in the lower mantle, it tends to metamorphose to perovskite, a mineral that requires a 3^+^ valence metal, normally aluminum. However, as the concentrations of Al^3+^ in the mantle are too low to meet this entire need, iron in the olivine disproportionates, with Fe^3+^ deputizing for the lacking Al^3+^, while the native iron Fe^0^ tends to gravitate to the core [61,62,63,85]. The result is a relatively oxidized volatisphere comprising CO_2_ > H_2_O >> N_2_ [85,124,125,126,127,128,129,130,131,132,133,134,135,136,137,138,139]. 

## 8. The Retreat to Mars! 

Some of the proponents of the ‘RNA-world hypothesis’ who recognize the geological, geophysical, isotopic and magmatic evidence for the early Earth being a “water world”, have retreated to Mars for their favored subaerial intermontane valleys assumed to have sheltered lakes subjected to wet-dry cycling [18,258,259,260,261]. According to this view, such valleys would have received high pH run-off from a watershed rich in serpentinizing olivines and eroding borate minerals in which to cosset and cook their organic soups. As water evaporated, “nucleobases, formylated nucleobases, and formylated carbohydrates, including formylated ribose, can form”(*sic*). We are then assured that “well-known chemistry transforms these structures into nucleosides, nucleotides, and partially formylated oligomeric RNA” [18]. Life that so emerged there was then distributed through a local panspermia to the otherwise deserted oceans of the early Earth. To our mind, this is the one speculative example where water would have been the enemy!

This whole idea of panspermia as an explanation for the “origin” of life on Earth was first given credence by no less than Hermann von Helmholtz in 1871 [262]—a suggestion provoking this scolding (in absentia!) from Karl Marx in 1875 [263]: “Helmholtz disseminated the absurd doctrine that the germs of terrestrial life fall ready-made from the moon, i.e., that they were brought down here by aerolites. I detest the kind of explanation which solves a problem by consigning it to some other locality”. 

## 9. Experimental Results Pertinent to the AVT

How is the AVT faring in the face of Sutherland’s [4] assumption that a “requirement for ultraviolet irradiation to generate hydrated electrons would rule out deep sea environments”? He continues “This, along with strong bioenergetic and structural arguments, suggests that the idea that life originated at vents should, like the vents themselves, remain ‘in the deep bosom of the ocean buried’.” We disposed of this fallacy in Section 2, and subject it to thermodynamic interrogation in Section 7. In Table 2, we list the experiments that have been applied to the AVT and their various outcomes since its first airing [36]. The AVT was not a passing whim to appear fully formed as that “pond” did in one of Darwin’s unguarded musings in that letter to Hooker. It had its own testing from its accidental conception, through a 30 year period of gestation beginning with employment in the chemical industry, although the actual form it took on its delivery in 1989 could not have been guessed (Table 2) [36,264]. The basis of submarine AVT is that the environment can support the continuous synthesis of large populations of monomers, encapsulating them in compartments which permit the formation of polymers of catalytic length. The current experimental focus of the submarine alkaline hydrothermal vent theory is to utilize free energy gradients for the synthesis and metabolic engagement of small organic molecules and monomers, which are precursors to biochemical processes. Further, the necessary disposal of waste is taken care of by direct hydrothermal expulsion in the ocean [5,47,72]. 

So, what are the AVT’s successes? To reiterate Russell [72]: the AHV theory did effectively predict the presence of off-ridge alkaline vents in the present oceans, a prognosis met by the discovery of the Lost City submarine alkaline vents in 2000 [36,146,265]. It also explains, for example, why early life did not have to invent such a counterintuitive mechanism as that entailed in Mitchell’s proton motive force to drive phosphate condensation (the only theory so to do) [142,266], how it was supplied with the necessary low entropy C1 feed [36,162,267], how biosynthesis could proceed in a highly radiated and mildly oxidized atmosphere [119], and why it was not destroyed by surface catastrophes in the Hadean” [95]. Since then, a microfluidics experiment by Hudson et al. [225] has demonstrated the reduction of CO_2_ to formate in a pH gradient, a key prediction of AVT. However, a natural proton motive force does not appear to have been the driver, and such a demonstration remains to be realized. We summarize other experimental results to be expected of the AVT in Table 2. 

## 10. How Might the Nucleotide Penthouse be Accessed from the Submarine Alkaline Vent

In a masterly critique of an article by Avshalom Elitzur [308], Yockey [34] muses on why the “primordial soup” hasn’t yielded the RNA world. This search, he suggests “seems to have been left for later in the manner of an ingenious architect in the Grand Academy of Lagado, as reported by Captain Lemuel Gulliver in Jonathan Swift’s Gulliver’s Travels. This architect contrived a new method for building houses by starting at the roof and working down and establishing the foundation at the end of the project. The architect pointed out that among the obvious advantages of this method is that once the roof was in place the workers could toil in the shade of the hot sun and at other times be protected from rain and snow. Thus, the progress of the construction would not be delayed by inclement weather. Although this idea had been approved by peer review, it was still in the research stage and he had not yet put in into practice at the time of Captain Gulliver’s visit.” Yockey continues; “following the reasoning of the architect in the Grand Academy of Lagado, cites the existence of life as a justification and a proof that a primeval soup must have existed.” Further, ‘‘the model proposed (of the origin of life) here is based on a simple assumption, namely, that life began with the accidental assembly of a self-replicating molecule (in a primeval soup). From this assumption the emergence of life naturally follows, enabling a new understanding of evolution as a whole. Thus, Elitzur and others are not deterred in their beliefs by the fact that the absence of evidence is indeed evidence of absence” [34]. 

All the geological and geochemical evidence demonstrates that the RNA world’s required ingredients for the Damer–Deamer soup; lipids, HCN, CH_3_N, H_2_S, H_2_O_2_, quinones, ferricyanide soup [9] simply weren’t available, and those sought by Bonfio and Mansy [66] such as acrolein, 2-aminooxazol, RNA, DNA, NADH and FMN, were even more outlandish. However, could the “submarine geyser help”? Duval and collaborators [185] point out that condensation of two amino or azanyl radicals will produce hydrazine in the interlayers of a hydrotalcite such as green rust. Hydrazine is an excellent feedstock for production of pyrazoles and imidazoles and other heterocyclic compounds—staging molecules for the nucleobases and the organic enzymes [185]. 

With this in mind, we compare and contrast the pond theory with the AVT in terms of putatively available “free energies” in Table 3. 

## 11. The “Origin of Life” Community

One of the inhibiting factors for the “origin of life” community is a general reluctance to accept that the emergence of life is a transdisciplinary, *hard* problem. Thereby, there is a tendency to ignore research disciplines outside of the main interests of the researchers themselves. Two significant disciplines that most researchers have an aversion to are those of geology and statistical thermodynamics. In this contribution, we have attempted to explain the geologic conditions at, and for, life’s emergence. For Boltzmannian thermodynamics as it applies to the AVT, and how pond theorists have failed to come to terms with it, the reader is referred to references [5,47,71,221,228].

## 12. What’s Next for the AVT?

None of the above criticisms of pond theory in this polemic should be taken to imply that the AVT has no serious issues or research challenges of its own. First amongst these is whether partially sulfurized green rust/fougerite was literally the first seed of life—exploited by the local disequilibria as a ‘makeshift’ protocell to enable their dissipation [72]—or was it merely coopted by peptides generated in the same environment along with iron sulfide—synthesized on site to be exploited as the first multi-tasking proto-enzyme, (or, of course, was it involved at all) [185]? Either way, many of the research challenges for the hypothesized role(s) of fougerite—dosed with various trace elements and anions—are similar. Such research addressing the submarine acid v. alkaline milieu calls for the further employment of tried-and-tested microfluidic and nano-crystallographic techniques [192,201,202,203,204,205,206,207,224,254,296,297,298,299,300,301,302,310,327,328,329,330,331,332,333,334,335,336,337,338]. We enumerate some possible developments from, expectations of, and tests for, the AVT below:

1. Can the fougerite/green rust interlayers—already shown to effect the relatively rapid eight electron reduction of nitrate to ammonia through-edge inward oxidation—be recharged from electrons generated at a transition-metal-rich hydrothermal vent (acting as a hydrogenase) through iron-to-iron hopping along the green rust metal oxide layers; i.e., is the green rust battery rechargeable at the vent [72,158,199,324,328,336,337,338,339]?

2. While green rust has been shown to be capable of aminating carboxylic to amino acids [231,340], the next vital and major challenge for the AVT is for a demonstration of condensations of amino acids to short peptides. 

3. Could an NO intermediate, produced from nitrite at Fe sites within the interlayers of fougerite, oxidize methane to a methyl group [267,306] cf. methane monooxygenase and the α-Fe/α-O active site in Fe-CHA zeolite [307]?

4. What proportion of the chemical transformations produced within green rust interlayers is the result of electrostatic forces and what is due to directional stresses and, anyway, are the two coupled [71,72,314,321,322,323]?

5. Further, are there analogies to be had, for example, between the electrostatic and conformational changes during polaron migration within the green rust interlayers to be expected during continuous reductions of nitrate and nitrite, with the changing dimensions of the Fe-N site in nitrite reductase [193,194,230,324,338,341]? 

6. Do Fe^3+^ polarons in general act to pump anions nano-peristaltically into and/or through the green rust interlayers, as well as pump nutrients through, and toxins and uncooperative molecular waste out of, the system [6,72,315,316,317,318,324,338]?

7. In the same vein, can low pH (local acidity) drive the condensation of orthophosphate to pyrophosphate to high disequilibria at the edges (binding sites) of fougerite galleries where the entropic state and water activity are low in the manner to be expected of the core of bioenergetics [310]? If so, can immediate hydrolyses leverage trapping of condensation reactions at neighboring (and oscillating) binding sites (cf. certain pyrophosphatases), i.e., can ‘macromolecular’ green rust effect alternating independent coupling as in the binding change mechanisms that are known to operate in enzymes such as the proton pyrophosphatases [72,228,315,316,317,318]?

8. Would a similar process result in the condensation of NH_2_ radicals to (N_2_H_4_) hydrazine, a step to heterocyclic redox molecules and the nucleotide world [185]?

9. Can the putative escapement mechanisms and information ratchets in the first green rust/fougerite nanoengines of life referred to above, work to produce the asymmetry and the irreversibility in a system necessary for life’s emergence—it’s climbing the steps that’s hard [2,6,72,318,319,320,321,322,323]?

10. In AVT, information transfer would have emerged coupled to protometabolism “in materio” in the green rust/fougerite interlayers: a fertile research area that begins to converge with research in emergence of intrinsic computing, nanoscience and nanotechnology [1,34,215,216,311,334,342,343,344,345,346,347,348,349,350,351,352,353,354,355,356,357,358,359,360].

## Figures and Tables

**Table 1:** A Hadean Advisory.

Effects	Descriptions	References
Solar luminosity post solar wind	72% of present flux	[75]
Solar radiation (UV and X-ray)	Intense: ~100 + times present	[76,77]
Earth–Moon distance; Earth’s spin and length of day and tides	<10% of present day; Estimates of length of day from 2 to 10 h; ~20 m tidal amplitude	[78,79,80,81,82,83,84,85,86,87,88,89,90,91]
Bombardment and tsunamis	Heavy but not totally vaporizing (negative feedback from heightening atmospheric pressure)	[92,93,94,95,96,97]
Maximum height of ocean plateaux above by mantle plumes.	~1000 km	[98,99,100,101,102,103,104,105,106,107]
Ocean depth	4–6 km	[108,109,110,111,112,113,114,115,116,117]
Redox state	Upper mantle buffered at quartz-fayalite-magnetite	[62,63,118,119,120]
Atmosphere post solar wind	CO_2_, N_2,_ H_2_O, > SO_2_ >> CO, NOx	[121,122,123,124,125,126,127,128,129,130,131,132,133]
Ocean chemistry	Saline, CO_2_, NO_3_^−^, NO_2_^−^ + metal ions	[134,135,136,137,138,139,140]
Magma-driven submarine springs	Acidic, ~400 °C	[141]
Direct contribution of ~400 °C solution to Hadean Ocean	Fe^2+^, Mg^2+^, Mn^2+^, Zn^2+^, Co^2+^, Ni^2+^, H_2_S, H_2_, PO_4_^−^, CH_4_	[142]
Serpentinization-driven submarine springs	Alkaline, ~120 °C	[143,144,145,146,147]
Direct contribution of ~120 °C alkaline spring to the hydrothermal mound	H_2_, CH_4_, HS^−^, HCOO^−^ > [Fe_2_S_2_(MoS_4_)_2_]^2−/4−^	[142,143,144,145,146,147,148,149]
Ocean T & pH and chemistry	Strongly carbonic and saline 25 to <85 °C? pH 4.5 to 5.5 with minor nitrate, transition metals in solution fed from ~400 °C springs	[129,141,150,151,152,153,154,155]
The Earth electronic and protonic ~1 volt battery	Eh of H_2_ v. H_2_O at delta pH 4 to 5	[136,156,157,158,159]
Olivine source of pyrophosphate delivered to ocean via vulcanism	Hydrolysis of volcanic P_4_O_10_ to produce P_3_O_9_^3−^ and P_4_O_12_^4−^	[160,161,162]
Lightning	Produces NO from CO_2_ + N_2_	[131,163,164,165,166,167,168,169]
Wind speed (cf. “Roaring Forties”)	12 ms^–1^ estimate	[170]
Wave height	10 m estimate	[86,170]
Chemical sediments	Banded iron formation, fougerite, chert, greenalite, mackinawite	[171,172,173,174,175,176,177,178,179]

**Table 2:** An AVT status report.

Prediction/Expectation	References	Tests	References
Hydrothermal circulation during rift tectonics generates metal sulfide mineral deposits.	[268]	Successful field test, discovery of giant base metal deposit, Navan, Ireland	[269,270]
Seawater-derived Downward hydrothermal convection driven by crustal heat and exothermic reactions	[271,272,273,274]	Stratigraphic, structural, tectonic and lithochemical field work; Lead isotope analyses	[275,276,277]
Some hydrothermal minerals precipitate on sea-floor	[269,270,271,272,273]	Delineation of extensive Mn aureole centered on Irish ore deposit led to the first discovery of fossil hydrothermal chimneys formed through mixing with seawater	[277,278,279,280,281,282]
Some sulfur derived from crustal sources	[157,273,283]	Isotopic analysis reveals crustal source as do hydrothermal experiments	[196,197,276]
Sulfide dissolves in alkaline hydrothermal solution	[157]	Lab demonstrations	[196,197,225]
Serpentinization reaction to formate	[284,285,286,287,288,289,290,291]	Lab demonstration	[226]
Serpentinization reaction produces H_2_ >>CH_4_, although CH_4_ is entrained from oceanic crust	[292]	Prompts amendment to the AVT, viz., to the denitrifying methanotrophic acetogenesis	[267]
Lightning and space weather radiation produces NOx that rapidly dissolve as nitrate/nitrite in Hadean Ocean	[126]	Theoretic studies generally support this hypothesis though not those of Ranjan et al. 2019 [286]	[131]
Source of ammonia at vent from nitrate/nitrite reduction	[126,163]	Eight electron reduction of nitrate to ammonia with green rust	[193,194,230]
Further reduction of NOx to hydrazine N_2_H_4_	[80]	Awaiting test	
Off-ridge submarine vents will be moderate temperature, H_2_-bearing, alkaline and long-lived (≥10^5^ years) and would have been the site of life’s emergence	[36,136,157,264,293]	Discovery of Lost City moderate temperature alkaline hydrothermal vent in North Atlantic Ocean	[146,265]
Green rust, mackinawite/greigite, amorphous silica barrier/ membrane	[36,39,72,294]	Successful lab demonstration	[36,159,197,232,251,295]
Eh and pH gradients ~700 mV and 4–5 units pH (~300 mV) to meet electronic and protonic requirements ~1 V	[157]	FeS barriers hold a 700 mV and a 5 unit pH disequilibrium in lab test	[199,231,254]
CO_2_ reduction forced by H_2_ and delta pH 4 units	[157]	Chemical disequilibria as per Nernst equation	[226]
The immateriality of the “water problem” in nano-confinement	[72,296,297,298,299,300,301,302,303,304,305]	Lab and molecular dynamic simulations	[201,202]
Aminations of carboxylic acids	[72]	Lab demonstrations. Amination of pyruvate to alanine	[231]
Green rust as proto- pyrophosphatase	[228]	Pi + Pi → PPi to equilibrium in microfluidic reactor	[254]
ΔpH as *pmf*	[63,156,157]	Undemonstrated	
Oxidation of methane in green rust	[72,267,306]	Undemonstrated, pending	Cf. [307]
Theoretical polymerization of amino acids in nano-confined water to produce peptide membranes necessarily pre-LUCA	[72]	Undemonstrated, waiting experiment	
Expansion from the vent via ocean floor to produce the first deep biosphere	[156]	Hypothesis	

**Table 3:** Pond and AVT chemistry and “free energies” compared.

	Surface Pond	References	Submarine Alkaline Vent	References
“Free energy”	UV, reverse 2 pH unit pmf	[7,16,59,60,68]	Electrochemical gradients, natural 4 pH unit pmf	[156,157,199,219,226,231,254,291]
Electron donors	UV radiation, reduced sulfur & organic compounds, Fe^2+^	[3,10,30,65]	Fe^2+^, H_2_, ē, CH_4_, HCOO^-^	[5,36,39,63,72,156,157,247,248,267,284,291,306,309,310]
Electron acceptors	Ferricyanide insidevesicle	[9]	Ambient Fe^3+^, NO, NO_2_^−^, NO_3_^−^ (CO_2_)	[39,131,157,166,291]
Initial boundary	Lipids	[7,49,51,64]	Green rust, FeS, silica	[36,37,38,39,72,157,291]
Organic takeover	Lipids	[7,9]	Peptides	[181,184,185,190,191,192,215,216,237,311]
Primary ingredients	Lipids HCN, CH_3_N, H_2_S, H_2_O_2_, quinones, ferricyanide	[9]	CO_2_, H_2_, CH_4_, H_2_O, NO_3_, NO, NH_2_, NH_3_, HPO_4_^3−^, HS^−^, Fe^2+^, Ni^2+^, Co^2+^, Mo^4+/6+^	[116,129,130,131,132,133,134,135,136,137,138,143,196,197,225,291,312]
Other suggestions orderivatives	Acrolein, 2-aminooxazol, quinones, ferricyanide, RNA, DNA, NADH, FMN	[11,16,30,60,64,65,66,67]	NH_3_ + carboxylic acids → amino acids, N_2_H_4_ → heterocycles, e.g., pyrazoles, imides, NAD(P), flavins, quinones	[185,193,194,230,310]
Disequilibria conversion mechanisms	Wet/dry cycling aggregation	[7]	Visco-elastic allosteric conformational changes/binding change mechanism/pumping/gating/ electrostatic effects	[5,47,71,72,181,182,183,184,185,186,187,188,189,190,191,192,221,228,310,313,314,315,316,317,318,319,320,321,322,323,324]
Condensations	Wet/dry cycling	[9,20,21,24,57]	Nanoconfined water in green rust interlayers, silica, mackinawite	[72,80,88,89,90,96,97,100,101,102,103,104,123,125,302]
Reproduction	RNA world	[9,17,22,51,64]	Amyloid peptide	[72,211,212,213,214,215,216,325,326]
Waste disposal	None considered	---	In alkaline spring effluent	[36,47,72]

## References

[1] Wicken J.S. (1987). Evolution, Thermodynamics and Information: Extending the Darwinian Program.

[2] Cottrell A. (1979). The natural philosophy of engines. Contemp. Phys..

[3] Marshall M. (2020). How the first life on Earth survived its biggest threat—water. Nature.

[4] Sutherland J.D. (2017). Studies on the origin of life—The end of the beginning. Nat. Rev. Chem..

[5] Branscomb E., Russell M.J. (2018). Frankenstein or a submarine alkaline vent: Who is responsible for abiogenesis? Part 2: As life is now, so it must have been in the beginning?. BioEssays.

[6] Milshteyn D., Damer B., Havig J.R., Deamer D. (2018). Amphiphilic Compounds Assemble into Membranous Vesicles in Hydrothermal Hot Spring Water but Not in Seawater. Life.

[7] Rajamani S., Vlassov A., Benner S., Coombs A., Olasagasti F., Deamer D. (2008). Lipid-assisted Synthesis of RNA-like Polymers from Mononucleotides. Orig. Life Evol. Biosph..

[8] Deamer D., Damer B., Kompanichenko V. (2019). Hydrothermal Chemistry and the Origin of Cellular Life. Astrobiology.

[9] Damer B., Deamer D. (2020). The Hot Spring Hypothesis for an Origin of Life. Astrobiology.

[10] Frenkel-Pinter M., Haynes J.W., Martin C., Petrov A.S., Burcar B.T., Krishnamurthy R., Hud N.V., Leman L.J., Williams L.D. (2019). Selective incorporation of proteinaceous over nonproteinaceous cationic amino acids in model prebiotic oligomerization reactions. Proc. Natl Acad. Sci. USA.

[11] Bonfio C., Russell D.A., Green N.J., Mariani A., Sutherland J.D. (2020). Activation chemistry drives the emergence of functionalised protocells. Chem. Sci..

[12] Schwartz A.W., Van der Veen M., Bisseling T., Chittenden G.J.F. (1973). Prebiotic phosphorylation. II-nucleotide synthesis in the reaction system apatite-cyanogen-water. BioSystems.

[13] Lahav N., Chang S. (1976). The possible role of solid surface area in condensation reactions during chemical evolution: Re-evaluation. J. Mol. Evol..

[14] Lahav N., White D., Chang S. (1978). Peptide formation in the prebiotic era: Thermal condensation of glycine in fluctuating clay environments. Science.

[15] Darwin F. (1888). The Life and Letters of Charles Darwin.

[16] Powner M.W., Gerland B., Sutherland J.D. (2009). Synthesis of activated pyrimidine ribonucleotides in prebiotically plausible conditions. Nature.

[17] Mulkidjanian A., Bychkov A., Dibrova D., Galperin M., Koonin E. (2012). Origin of first cells at terrestrial, anoxic geothermal fields. Proc. Natl. Acad. Sci. USA.

[18] Benner S.A., Kim H.J., Carrigan M.A. (2012). Asphalt, water, and the prebiotic synthesis of ribose, ribonucleosides, and RNA. Acc. Chem. Res..

[19] Hud N.V. (2018). Searching for lost nucleotides of the pre-RNA World with a self-refining model of early Earth. Nat. Commun..

[20] Ross D.S., Deamer D. (2016). Dry/Wet Cycling and the Thermodynamics and Kinetics of Prebiotic Polymer Synthesis. Life.

[21] Damer B., Deamer D. (2015). Coupled phases and combinatorial selection in fluctuating hydrothermal pools: A scenario to guide experimental approaches to the origin of cellular life. Life.

[22] Pearce B.K.D., Pudritz R.E., Semenov D.A., Henning T.K. (2017). Origin of the RNA world: The fate of nucleobases in warm little ponds. Proc. Natl. Acad. Sci. USA.

[23] Becker S., Schneider C., Okamura H., Crisp A., Amatov T., Dejmek M., Carell T. (2018). Wet-dry cycles enable the parallel origin of canonical and non-canonical nucleosides by continuous synthesis. Nat. Commun..

[24] Becker S., Feldmann J., Wiedemann S., Okamura H., Schneider C., Iwan K., Crisp A., Rossa M., Amatov T., Carell T. (2019). Unified prebiotically plausible synthesis of pyrimidine and purine RNA ribonucleotides. Science.

[25] Hargrave M., Spencer S.K., Deamer D.W. (2018). Computational models of polymer synthesis driven by dehydration/ rehydration cycles: Repurination in simulated hydrothermal fields. J. Mol. Evol..

[26] Hargreaves W.R., Mulvihill S.J., Deamer D.W. (1977). Synthesis of phospholipids and membranes in prebiotic conditions. Nature.

[27] Nainytė M., Müller F., Ganazzoli G., Chan C.Y., Crisp A., Globisch D., Carell T. (2020). Amino Acid Modified RNA Bases as Building Blocks of an Early Earth RNA-Peptide World. Chemistry.

[28] Benner S.A., Bell E.A., Biondi E., Brasser R., Carell T., Kim H.J., Mojzsis S.J., Omran A., Pasek M.A., Trail D. (2019). When did life likely emerge on Earth in an RNA-first process?. arXiv.

[29] Damer B.F. (2016). A field trip to the Archaean in search of Darwin’s warm little pond. Life.

[30] Clark B.C., Kolb V.M. (2018). Comet Pond II: Synergistic Intersection of Concentrated Extraterrestrial Materials and Planetary Environments to Form Procreative Darwinian Ponds. Life.

[31] Van Kranendonk M.J., Baumgartner R., Djokic T., Ota T., Steller L., Garbe U., Nakamura E. (2021). Elements for the Origin of Life on Land: A Deep-Time Perspective from the Pilbara Craton of Western Australia. Astrobiology.

[32] Van Kranendonk M.J., Deamer D.W., Djokic T. (2017). Life springs: Darwin’s warm little pond revisited. Sci. Am..

[33] Darwin C. (1872). The Origin of Species.

[34] Yockey H.P. (1995). Comments on “Let there be life”; Thermodynamic reflections on biogenesis and evolution” by Avshalom C. Elitzur. J. Theor. Biol..

[35] Hud N., Brian J., Cafferty B.J., Krishnamurthy R., Williams R.D. (2013). The Origin of RNA and ‘‘My Grandfather’s Axe’’. Chem. Biol..

[36] Russell M.J., Hall A.J., Turner D. (1989). In vitro growth of iron sulphide chimneys: Possible culture chambers for origin-of-life experiments. Terra Nova.

[37] Russell M.J., Brown C.G., Hawkesworth C.J., Wilson R.C.L. (1992). Plate tectonics and hydrothermal ore deposits. Understanding the Earth.

[38] Russell M.J., Hall A.J., Fallick A.E., Boyce A.J. (2005). On hydrothermal convection systems and the emergence of life. Econ. Geol..

[39] Nitschke W., Russell M.J. (2009). Hydrothermal focusing of chemical and chemiosmotic energy, supported by delivery of catalytic Fe, Ni, Mo/W, Co, S and Se, forced life to emerge. J. Mol. Evol..

[40] Shapiro R. (1986). Origins: A Skeptic’s Guide to the Creation of Life on Earth.

[41] Lazcano A., Miller S.L. (1999). On the origin of metabolic pathways. J. Mol. Evol..

[42] Dass A.V., Hickman-Lewis K., Brack A., Kee T.P., Westall F. (2016). Stochastic prebiotic chemistry within realistic geological systems. ChemistrySelect.

[43] Guttenberg N., Virgo N., Chandru K., Scharf C., Mamajanov I. (2017). Bulk measurements of messy chemistries are needed for a theory of the origins of life. Philos. Trans. R. Soc. A.

[44] Walker S.I., Bains W., Cronin L., DasSarma S., Danielache S., Domagal-Goldman S., Kacar B., Kiang N.Y., Lenardic A., Reinhard C.T. (2018). Exoplanet biosignatures: Future directions. Astrobiology.

[45] Schrödinger E. (1945). What is Life? The Physical Aspect of the Living Cell; Based on Lectures Delivered under the Auspices of the Institute at Trinity College, Dublin, in February.

[46] Prigogine I., Jantsch E. (1976). Order through fluctuations: Self-organization and social systems. Evolution and Consciousness: Human Systems in Transition.

[47] Branscomb E., Russell M.J. (2018). Frankenstein or a submarine alkaline vent: Who is responsible for abiogenesis? Part 1: What is life—that it might create itself?. BioEssays.

[48] Hamilton W. (1855). Discussions on Philosophy and Literature.

[49] Deamer D.W., Barchfeld G.L. (1982). Encapsulation of macromolecules by lipid vesicles under simulated prebiotic conditions. J. Mol. Evol..

[50] Monnard P.A., Apel C.L., Kanavarioti A., Deamer D.W. (2002). Influence of ionic inorganic solutes on self-assembly and polymerization processes related to early forms of life: Implications for a prebiotic aqueous medium. Astrobiology.

[51] Deamer D., Singaram S., Rajamani S., Kompanichenko V., Guggenheim S. (2006). Self-assembly processes in the prebiotic environment. Phil. Trans. R. Soc. B.

[52] Mulkidjanian A.Y. (2009). On the origin of life in the zinc world: Photosynthesizing, porous edifices built of hydrothermally precipitated zinc sulfide as cradles of life on Earth. Biol. Dir..

[53] Mulkidjanian A.Y., Cherepanov D.A., Galperin M.Y. (2003). Survival of the fittest before the beginning of life: Selection of the first oligonucleotide-like polymers by UV light. BMC Evol. Biol..

[54] Deamer D., Weber A.L. (2010). Bioenergetics and life’s origins. Cold Spring Harbor Perspect. Biol..

[55] Hazen R.M., Sverjensky D.A. (2010). Mineral surfaces, geochemical complexities, and the origins of life. Cold Spring Harbor Perspect. Biol..

[56] Kim H.J., Ricardo A., Illangkoon H.I., Kim M.J., Carrigan M.A., Frye F., Benner S.A. (2011). Synthesis of carbohydrates in mineral-guided prebiotic cycles. J. Am. Chem. Soc..

[57] De Guzman V., Shenasa H., Vercoutere W., Deamer D. (2014). Generation of oligonucleotides under hydrothermal conditions by non-enzymatic polymerization. J. Mol. Evol..

[58] Forsythe J.G., Yu S.S., Mamajanov I., Grover M.A., Krishnamurthy R., Fernández F.M., Hud N.V. (2015). Ester-mediated amide bond formation driven by wet–dry cycles: A possible path to polypeptides on the prebiotic Earth. Angew. Chem. Int. Ed. Engl..

[59] Hazen R.M. (2017). Chance, necessity and the origins of life: A physical sciences perspective. Philos. Trans. R. Soc. A.

[60] Ritson D., Sutherland J.D. (2012). Prebiotic synthesis of simple sugars by photoredox systems chemistry. Nat. Chem..

[61] Shock E.L. (1992). Chemical environments of submarine hydrothermal systems. Orig. Life Evol. Biosph..

[62] Wood B.J., Walter M.J., Wade J. (2006). Accretion of the Earth and segregation of its core. Nature.

[63] Russell M.J., Ponce A. (2020). Six ‘Must-Have’ Minerals for Life’s Emergence: Olivine, Pyrrhotite, Bridgmanite, Serpentine, Fougerite and Mackinawite. Life.

[64] Patel B.H., Percivalle C., Ritson D.J., Duffy C.D., Sutherland J.D. (2015). Common origins of RNA, protein and lipid precursors in a cyanosulfidic protometabolism. Nat. Chem..

[65] Xu J., Chmela V., Green N.J., Russell D.A., Janicki M.J., Góra R.W., Szabla R., Bond A.D., Sutherland J.D. (2020). Selective prebiotic formation of RNA pyrimidine and DNA purine nucleosides. Nature.

[66] Bonfio C., Godino E., Corsini M., de Biani F.F., Guella G., Mansy S.S. (2018). Prebiotic iron–sulfur peptide catalysts generate a pH gradient across model membranes of late protocells. Nat. Catal..

[67] Bonfio C., Valer L., Scintilla S., Shah S., Evans D.J., Jin L., Szostak J.W., Sasselov D.D., Sutherland J.D., Mansy S.S. (2017). UV-light-driven prebiotic synthesis of iron–sulfur clusters. Nat. Chem..

[68] Mann A. (2021). Inner Workings: Making headway with the mysteries of life’s origins. Proc. Natl. Acad. Sci. USA.

[69] Deamer D. (2017). Conjecture and hypothesis: The importance of reality checks. Beilst. J. Org. Chem..

[70] Popper K. (1963). Conjectures and Refutations. The Growth of Scientific Knowledge.

[71] Branscomb E., Biancalani T., Goldenfeld N., Russell M. (2017). Escapement mechanisms and the conversion of disequilibria; the engines of creation. Phys. Rep..

[72] Russell M.J. (2018). Green Rust: The Simple Organizing ‘Seed’ of All Life?. Life.

[73] Burcar B., Pasek M., Gull M., Cafferty B.J., Velasco F., Hud N.V., Menor-Salván C. (2016). Darwin’s warm little pond: A one-pot reaction for prebiotic phosphorylation and the mobilization of phosphate from minerals in a urea-based solvent. Angew. Chem. Int. Ed. Engl..

[74] Deamer D. (2021). Where Did Life Begin? Testing Ideas in Prebiotic Analogue Conditions. Life.

[75] Bahcall J.N., Pinsonneault M.H., Basu S. (2001). Solar models: Current epoch and time dependences, neutrinos, and helioseismo-logical properties. Astrophys. J..

[76] Pipin V.V., Kosovichev A.G. (2015). Effects of large-scale non-axisymmetric perturbations in the mean-field solar dynamo. Astrophys. J..

[77] Gudel M. (2007). The sun in time: Activity and environment. Living Rev. Sol. Phys..

[78] Birch F. (1965). Energetics of core formation. J. Geophys. Res..

[79] Longuet-Higgins M.S. (1968). The eigenfunctions of Laplace’s tidal equation over a sphere. Philos. Trans. R. Soc. Lond. A.

[80] Binder A.B. (1982). The Moon: Its figure and orbital evolution. Geophys. Res. Lett..

[81] Dones L., Tremaine S. (1993). Why Does the Earth Spin Forward?. Science.

[82] Zharkov V.N. (2000). On the history of the lunar orbit. Astron. Vesn..

[83] Denis C., Rybicki K.R., Schreider A.A., Tomecka-Suchoń S., Varga P. (2011). Length of the day and evolution of the Earth’s core in the geological past. Astron. Nachr..

[84] Denis C., Schreider A.A., Varga P., Zavoti J. (2002). Despinning of the Earth rotation in the geological past and geomagnetic paleointensities. J. Geodyn..

[85] Sossi P.A., Burnham A.D., Badro J., Lanzirotti A., Newville M., O’Neill H.S.C. (2020). Redox state of Earth’s magma ocean and its Venus-like early atmosphere. Sci. Adv..

[86] Dandonneau Y., Vega A., Loisel H., Du Penhoat Y., Menkes C. (2003). Oceanic Rossby waves acting as a “hay rake” for ecosystem floating by-products. Science.

[87] Glukhovskii M.Z., Kuz’min M.I. (2015). Extraterrestrial factors and their role in the Earth’s tectonic evolution in the early Precambrian. Russ. Geol. Geophys..

[88] Marakushev A.A., Zinov’eva N.G., Paneyakh N.A., Marakushev S.A. (2013). The origin and evolution of the solar system. Prostran I Vremya.

[89] Malcuit R.J. (2015). A Retrograde Gravitational Capture Model for the Earth-Moon System. In The Twin Sister Planets Venus and Earth.

[90] Bozóki T., Herein M., Galsa A. (2017). Numerical evolution of the asymmetry in the compositionally inhomogeneous lower mantle driven by Earth’s rotation. Acta Geodaet. Geophys..

[91] Lingam M., Loeb A. (2018). Implications of tides for life on exoplanets. Astrobiology.

[92] Glikson A.Y. (2004). Early Precambrian asteroid impact-triggered tsunami: Excavated seabed, debris flows, exotic boulders, and turbulence features associated with 3.47–2.47 Ga-old asteroid impact fallout units, Pilbara Craton, Western Australia. Astrobiology.

[93] Abramov O., Mojzsis S.J. (2009). Microbial habitability of the Hadean Earth during the Late Heavy Bombardment. Nature.

[94] Sleep N.H. (2012). Maintenance of permeable habitable subsurface environments by earthquakes and tidal stresses. Int. J. Astrobiol..

[95] Abramov O., Kring D.A., Mojzsis S.J. (2013). The impact environment of the hadean earth. Geochemistry.

[96] Boehnke P., Harrison T.M. (2016). Illusory late heavy bombardments. Proc. Natl. Acad. Sci. USA.

[97] Zellner N.E. (2017). Cataclysm no more: New views on the timing and delivery of lunar impactors. Orig. Life Evol. Biosph..

[98] Monteux J., Andrault D., Guitreau M., Samuel H., Demouchy S. (2020). A mushy Earth’s mantle for more than 500 Myr after the magma ocean solidification. Geophys. J. Int..

[99] Solomatov V.S. (2007). Magma Oceans and Primordial Mantle Differentiation. Treat. Geophys..

[100] Walter M.J., Nakamura E., Trønnes R.G., Frost D.J. (2005). Experimental constraints on crystallization differentiation in a deep magma ocean. Geochim. Cosmochim. Acta.

[101] Sleep N.H., Zahnle K.J., Lupu R.E. (2014). Terrestrial after-math of the Moon-forming impact. Philos. Trans. R. Soc. A.

[102] Kamber B.S. (2015). The evolving nature of terrestrial crust from the Hadean, through the Archaean, into the Proterozoic. Precam. Res..

[103] Morgan J.P., Morgan W.J. (1999). Two-stage melting and the goldschmgeochemical evolution of the mantle: A recipe for mantle plum-pudding. Earth Planet. Sci. Lett..

[104] Şengör A.M.C., Ernst R.E., Buchan K.L. (2001). Elevation as indicator of mantle-plume activity. In Mantle plumes: Their identification through time. Geol. Soc. Am..

[105] Mann P., Taira A. (2004). Global tectonic significance of the Solomon Islands and Ontong Java Plateau convergent zone. Tectonophysics.

[106] Russell M.J., Arndt N.T. (2005). Geodynamic and metabolic cycles in the Hadean. Biogeosciences.

[107] Bédard J.H. (2006). A catalytic delamination-driven model for coupled genesis of Archaean crust and sub-continental lithospheric mantle. Geochim. Cosmochim. Acta.

[108] Morbidelli A., Chambers J., Lunine J.I., Petit J.M., Robert F., Valsecchi G.B., Cyr K.E. (2000). Source regions and timescales for the delivery of water to the Earth. Meteorit. Planet. Sci..

[109] Bounama C., Franck S., von Bloh W. (2001). The fate of the Earth’s ocean. Hydrol. Earth Syst. Sci..

[110] Valley J.W., Lackey J.S., Cavosie A.J., Clechenko C.C., Spicuzza M.J., Basei M.A.S., Bindeman I.N., Ferreira V.P., Sial A.N., King E.M. (2005). 4.4 billion years of crustal maturation: Oxygen isotope ratios of magmatic zircon. Contr. Miner. Petrol..

[111] Cavosie A.J., Valley J.W., Wilde S.A. (2007). The oldest terrestrial mineral record: A review of 4400 to 3900 ma detrital zircons from Jack Hills, Western Australia. Dev. Precamb. Geol..

[112] Pope E.C., Bird D.K., Rosing M.T. (2012). Isotope composition and volume of Earth’s early oceans. Proc. Natl. Acad. Sci. USA.

[113] O’Neil J., Boyet M., Carlson R.W., Paquette J.-L. (2013). Half a billion years of reworking of Hadean mafic crust to produce the Nuvvuagittuq Eoarchean felsic crust. Earth Planet. Sci. Lett..

[114] Korenaga J., Planavsky N.J., Evans D.A. (2017). Global water cycle and the coevolution of the Earth’s interior and surface environment. Phil. Trans. R. Soc. A.

[115] Genda H. (2016). Origin of Earth’s oceans: An assessment of the total amount, history and supply of water. Geochem. J..

[116] Ueda H., Shibuya T. (2021). Composition of the Primordial Ocean Just after Its Formation: Constraints from the Reactions between the Primitive Crust and a Strongly Acidic, CO_2_-Rich Fluid at Elevated Temperatures and Pressures. Minerals.

[117] Johnson B.W., Wing B.A. (2020). Limited Archaean continental emergence reflected in an early Archaean ^18^O-enriched ocean. Nat. Geosci..

[118] Deng J., Du Z., Karki B.B., Ghosh D.B., Lee K.K. (2020). A magma ocean origin to divergent redox evolutions of rocky planetary bodies and early atmospheres. Nat. Commun..

[119] Trail D., Watson E.B., Tailby N.D. (2011). The oxidation state of Hadean magmas and implications for early Earth’s atmosphere. Nature.

[120] Galimov E.M. (2005). Redox evolution of the Earth caused by a multi-stage formation of its core. Earth Planet. Sci. Lett..

[121] Lammer H., Stökl A., Erkaev N.V., Dorfi E.A., Odert P., Güdel M., Kulikov Y.N., Kislyakova K.G., Leitzinger M. (2014). Origin and loss of nebula-captured hydrogen envelopes from ‘sub’-to ‘super-Earths’ in the habitable zone of Sun-like stars. Mon. Not. R. Astron. Soc..

[122] Massol H., Hamano K., Tian F., Ikoma M., Abe Y., Chassefière E., Davaille A., Genda H., Güdel M., Hori Y. (2016). Formation and evolution of protoatmospheres. Space Sci. Rev..

[123] Zahnle K., Arndt N., Cockell C., Halliday A., Nisbet E., Selsis F., Sleep N.H. (2007). Emergence of a habitable planet. Space Sci. Rev..

[124] Yung Y.L., McElroy M.B. (1979). Fixation of nitrogen in the prebiotic atmosphere. Science.

[125] Kasting J.F., Ackerman T.P. (1986). Climatic consequences of very high carbon dioxide levels in the Earth’s early atmosphere. Science.

[126] Zhang X., Helsdon J.J.H., Farley R.D. (2003). Numerical modeling of lightning produced NOx using an explicit lightning scheme: Two-dimensional simulation as a ‘proof of concept’. J. Geophys. Res..

[127] Dasgupta R., Hirschmann M.M. (2006). Melting in the Earth’s deep upper mantle caused by carbon dioxide. Nature.

[128] Hirschmann M.M., Tenner T., Aubaud C., Withers A.C. (2009). Dehydration melting of nominally anhydrous mantle: The primacy of partitioning. Phys. Earth Planet. Inter..

[129] Hirschmann M.M. (2018). Comparative deep Earth volatile cycles: The case for C recycling from exosphere/mantle fractionation of major (H_2_O, C, N) volatiles and from H_2_O/Ce, CO_2_/Ba, and CO_2_/Nb exosphere ratios. Earth Planet. Sci. Lett..

[130] Martin R.S., Mather T.A., Pyle D.M. (2007). Volcanic emissions and the early earth atmosphere. Geochim. Cosmochim. Acta.

[131] Wong M.L., Charnay B.D., Gao P., Yung Y.L., Russell M.J. (2017). Nitrogen Oxides in Early Earth’s Atmosphere as Electron Acceptors for Life’s Emergence. Astrobiology.

[132] Gebauer S., Grenfell J.L., Lammer H., de Vera J.P., Sproß L., Airapetian V.S., Sinnhuber M., Rauer H. (2020). Atmospheric nitrogen when life evolved on Earth. Astrobiology.

[133] Catling D.C., Zahnle K.J. (2020). The archean atmosphere. Sci. Adv..

[134] Datz S., Smith W.T., Taylor E.H. (1961). Molecular association in alkali halide vapors. J. Chem. Phys..

[135] Van Groos A.K., Wyllie P.J. (1969). Melting relationships in the system NaAlSi_3_O_8_-NaCl-H_2_O at one kilobar pressure, with petrological applications. J. Geol..

[136] Macleod G., McKeown C., Hall A.J., Russell M.J. (1994). Hydrothermal and oceanic pH conditions of possible relevance to the origin of life. Orig. Life Evol. Biosph..

[137] Kusakabe M., Tanyileke G.Z., McCord S.A., Schladow S.G. (2000). Recent pH and CO_2_ profiles at Lakes Nyos and Monoun, Cameroon: Implications for the degassing strategy and its numerical simulation. J. Volcanol. Geotherm. Res..

[138] Kamenetsky M.B., Sobolev A.V., Kamenetsky V.S., Maas R., Danyushevsky L.V., Thomas R., Sobolev N.V., Pokhilenko N.P. (2004). Kimberlite melts rich in alkali chlorides and carbonates: A potent metasomatic agent in the mantle. Geology.

[139] Pinti D.L. (2005). The origin and evolution of the oceans. Lectures in Astrobiology.

[140] Hanley J.J., Mungall J.E., Pettke T., Spooner E.T.C., Bray C.J. (2008). Fluid and halide melt inclusions of magmatic origin in the Ultramafic and Lower Banded Series, Stillwater Complex, Montana, USA. J. Petrol..

[141] Kump L.R., Seyfried W.E. (2005). Hydrothermal Fe fluxes during the Precambrian: Effect of low oceanic sulfate concentrations and low hydrostatic pressure on the composition of black smokers. Earth Planet. Sci. Lett..

[142] German C.R., Von Damm K.L. (2003). Hydrothermal processes. Treatise Geochem..

[143] White L.M., Shibuya T., Vance S.D., Christensen L.E., Bhartia R., Kidd R., Hoffmann A., Stucky G.D., Kanik I., Russell M.J. (2020). Simulating serpentinization as it could apply to the emergence of life using the JPL hydrothermal reactor. Astrobiology.

[144] Seyfried W.E., Pester N.J., Tutolo B.M., Ding K. (2015). The Lost City hydrothermal system: Constraints imposed by vent fluid chemistry and reaction path models on subseafloor heat and mass transfer processes. Geochim. Cosmochim. Acta.

[145] Kelley D.S., Karson J.A., Blackman D.K., Früh-Green G.L., Butterfield D.A., Lilley M.D., Olson E.J., Schrenk M.O., Roe K.K., 1043 Lebon G.T. (2001). An off-axis hydrothermal vent field near the Mid-Atlantic Ridge at 30 N. Nature.

[146] Kelley D.S., Karson J.A., Früh-Green G.L., Yoerger D.R., Shank T.M., Butterfield D.A., Hayes J.M., Schrenk M.O., Olson E.J., Proskurowski G. (2005). A serpentinite-hosted ecosystem: The Lost City hydrothermal field. Science.

[147] Lowell R.P., Rona P.A. (2002). Seafloor hydrothermal systems driven by the serpentinization of peridotite. Geophys. Res. Lett..

[148] Lang S.Q., Brazelton W.J. (2020). Habitability of the marine serpentinite subsurface: A case study of the Lost City hydrothermal field. Philos. Trans. R. Soc. A.

[149] Rasmussen B., Muhling J.R., Fischer W.W. (2021). Greenalite Nanoparticles in Alkaline Vent Plumes as Templates for the Origin of Life. Astrobiology.

[150] Mojzsis S.J., Harrison T.M., Pidgeon R.T. (2001). Oxygen-isotope evidence from ancient zircons for liquid water at the Earth’s sur-face 4300 Myr ago. Nature.

[151] Knauth L.P. (2005). Temperature and salinity history of the Precambrian ocean: Implications for the course of microbial evolution. Palaeogeog. Palaeoclimat. Palaeoecol..

[152] Robert F., Chaussidon M. (2006). A palaeotemperature curve for the Precambrian oceans based on silicon isotopes in cherts. Nature.

[153] Fisher C.M., Vervoort J.D. (2008). Using the magmatic record to constrain the growth of continental crust—The Eoarchean zircon Hf record of Greenland. Earth Planet. Sci. Lett..

[154] Tartèse R., Chaussidon M., Gurenko A., Delarue F., Robert F. (2017). Warm Archean oceans reconstructed from oxygen isotope composition of early-life remnants. Geochem. Perspect. Lett..

[155] Greenwood R.C., Barrat J.A., Miller M.F., Anand M., Dauphas N., Franchi I.A., Sillard P., Starkey N.A. (2018). Oxygen isotopic evidence for accretion of Earth’s water before a high-energy Moon-forming giant impact. Sci. Adv..

[156] Russell M.J., Hall A.J. (2006). The onset and early evolution of life. Geol. Soc. Am..

[157] Russell M.J., Hall A.J. (1997). The emergence of life from iron monosulphide bubbles at a submarine hydrothermal redox and pH front. J. Geol. Soc. Lond..

[158] Yamamoto M., Nakamura R., Kasaya T., Kumagai H., Suzuki K., Takai K. (2017). Spontaneous and widespread electricity generation in natural deep-sea hydrothermal fields. Angew. Chem. Int. Ed. Engl..

[159] Russell M.J., Hall A.J., Mellersh A.R., Ikan R. (2003). On the dissipation of thermal and chemical energies on the early Earth: The onsets of hydrothermal convection, chemiosmosis, genetically regulated metabolism and oxygenic photosynthesis. Natural and Laboratory-Simulated Thermal Geochemical Processes.

[160] Yamagata Y., Watanabe H., Saitoh M., Namba T. (1991). Volcanic production of pyrophosphate and its relevance to prebiotic evolution. Nature.

[161] Milman-Barris M.S., Beckett J.R., Baker M.B., Hofmann A.E., Morgan Z., Crowley M.R., Vielzeuf D., Stolper E. (2008). Zoning of phosphorus in igneous olivine. Contr. Min. Petrol..

[162] Veter M., Foley S.F., Mertz-Kraus R., Groschopf N. (2017). Trace elements in olivine of ultramafic lamprophyres controlled by phlogopite-rich mineral assemblages in the mantle source. Lithos.

[163] Nishizawa M., Saito T., Makabe A., Ueda H., Saitoh M., Shibuya T., Takai K. (2021). Stable Abiotic Production of Ammonia from Nitrate in Komatiite-Hosted Hydrothermal Systems in the Hadean and Archean Oceans. Minerals.

[164] Sleep N.H. (2018). Geological and geochemical constraints on the origin and evolution of life. Astrobiology.

[165] Gurevich A.V., Zybin K.P. (2005). Runaway breakdown and the mysteries of lightning. Phys. Today.

[166] Ducluzeau A.-L., van Lis R., Duval S., Schoepp-Cothenet B., Russell M.J., Nitschke W. (2009). Was nitric oxide the first strongly oxidizing terminal electron sink?. Trends Biochem. Sci..

[167] Adelman Z. Technical Memorandum No. 12: Sea Salt and Lightning. June 25, 2012, 773 San Marin Drive, Suite 2115, No-vato, CA 94998 P: 415-899-0700 F: 415-899-0707. www.environcorp.com.

[168] Hu R., Diaz H.D. (2019). Stability of nitrogen in planetary atmospheres in contact with liquid water. Astrophys. J..

[169] Airapetian V.S., Barnes R., Cohen O., Collinson G.A., Danchi W.C., Dong C.F., Del Genio A.D., France K., Garcia-Sage K., Glocer A. (2020). Impact of space weather on climate and habitability of terrestrial-type exoplanets. Int. J. Astrobiol..

[170] Liu W.T. (2001). Wind over troubled water. Backscatter.

[171] Mloszewska A.M., Pecoits E., Cates N.L., Mojzsis S.J., O’Neil J., Robbins L.J., Konhauser K.O. (2012). The composition of Earth’s oldest iron formations: The Nuvvuagittuq Supracrustal Belt (Québec, Canada). Earth Planet. Sci. Lett..

[172] Appel P.W.U. (1980). On the early Archaean Isua iron-formation, west Greenland. Precamb. Res..

[173] Appel P.W.U. (1990). Mineral occurrences in the 3.6 Ga old Isua supracrustal belt, West Greenland. Develop. Precamb. Geol..

[174] Gäb F., Ballhaus C., Siemens J., Heuser A., Lissner M., Geisler T., Garbe-Schönberg D. (2017). Siderite cannot be used as CO_2_ sensor for Archaean atmospheres. Geochim. Cosmochim. Acta.

[175] Halevy I., Alesker M., Schuster E.M., Popovitz-Biro R., Feldman Y. (2017). A key role for green rust in the Precambrian oceans and the genesis of iron formations. Nat. Geosci..

[176] Isley A.E., Abbott D.H. (1999). Plume-related mafic volcanism and the deposition of banded iron formation. J. Geophys. Res. Solid Earth.

[177] Klein C. (2005). Some Precambrian banded iron-formations (BIFs) from around the world: Their age, geologic setting, mineralogy, metamorphism, geochemistry, and origins. Am. Mineral..

[178] Mojzsis S.J., Arrhenius G., McKeegan K.D., Harrison T.M., Nutman A.P., Friend C.R.L. (1996). Evidence for life on earth before 3,800 million years ago. Nature.

[179] Tosca N.J., Guggenheim S., Pufahl P.K. (2016). An authigenic origin for Precambrian greenalite: Implications for iron formation and the chemistry of ancient seawater. Geol. Soc. Am. Bull..

[180] Voosen P. (2021). Ancient Earth was a water world. Sci. Mag..

[181] Zhang S., Holmes T., Lockshin C., Rich A. (1993). Spontaneous assembly of a self-complimentary oligopeptide to form a stable macroscopic membrane. Proc. Natl. Acad. Sci. USA.

[182] Brack A., Orgel L.E. (1975). β structures of alternating polypeptides and their possible prebiotic significance. Nature.

[183] Brack A., Spach G. (1981). Multiconformational synthetic polypeptides. J. Am. Chem. Soc..

[184] Milner-White E.J., Russell M.J. (2008). Predicting the conformations of proteins and peptides in early evolution. Biol. Dir..

[185] Duval S., Baymann F., Schoepp-Cothenet B., Trolard F., Bourrié G., Grauby O., Branscomb E., Russell M.J., Nitschke W. (2019). Fougerite: The not so simple progenitor of the first cells. Interface Focus.

[186] Walker J., Saraste M., Runswick M.J., Gay N.J. (1982). Distantly related sequences in the alpha- and beta-subunits of ATP synthase, myosin, kinases and other ATP-requiring enzymes and a common nucleotide binding fold. EMBO. J..

[187] Saraste M., Sibbald P.R., Wittinghofer A. (1990). The P-loop—A common motif in ATP and GTP-binding proteins. Trends Biochem. Sci..

[188] Watson J., Milner-White E.J. (2002). A Novel Main-chain Anion-binding Site in Proteins: The Nest. J. Mol. Biol..

[189] Pal D., Sühnel J., Weiss M.S. (2002). New principles of protein structure: Nests, eggs and what next?. Angew. Chem. Int. Ed. Engl..

[190] Bianchi A., Giorgi C., Ruzza P., Toniolo C., Milner-White E.J. (2012). A synthetic hexapeptide designed to resemble a proteinaceous P-loop nest is shown to bind inorganic phosphate. Proteins.

[191] Pras M., Schubert M., Zucker-Franklin D., Rimon A., Franklin E.C. (1968). The characterization of soluble amyloid prepared in water. J. Clin. Investig..

[192] Argudo P.G., Giner-Casares J.J. (2021). Folding and self-assembly of short intrinsically disordered peptides and protein regions. Nanoscale Adv..

[193] Hansen H.C.B., Koch C.B. (1998). Reduction of nitrate to ammonium by sulphate green rust: Activation energy and reaction mechanism. Clay Min..

[194] Hansen H.C.B., Guldberg S., Erbs M., Koch C.B. (2001). Kinetics of nitrate reduction by green rusts—effects of interlayer anion and Fe (II): Fe (III) ratio. Appl. Clay Sci..

[195] Fan R., Huh S., Yan R., Arnold J., Yang P. (2008). Gated proton transport in aligned mesoporous silica films. Nat. Mat..

[196] Mielke R.E., Russell M.J., Wilson P.R., McGlynn S.E., Coleman M., Kidd R., Kanik I. (2010). Design, fabrication, and test of a hydrothermal reactor for origin-of-life experiments. Astrobiology.

[197] Mielke R.E., Robinson K.J., White L.M., McGlynn S.E., McEachern K., Bhartia R., Kanik I., Russell M.J. (2011). Iron-sulfide-bearing chimneys as potential catalytic energy traps at life’s emergence. Astrobiology.

[198] Emmez E., Yang B., Shaikhutdinov S., Freund H.J. (2014). Permeation of a single-layer SiO_2_ membrane and chemistry in confined space. J. Phys. Chem. C.

[199] Barge L.M., Abedian Y., Russell M.J., Doloboff I.J., Cartwright J.H., Kidd R.D., Kanik I. (2015). From Chemical Gardens to Fuel Cells: Generation of Electrical Potential and Current Across Self-Assembling Iron Mineral Membranes. Angew. Chem. Int. Ed. Engl..

[200] Silies L., Didzoleit H., Hess C., Stühn B., Andrieu-Brunsen A. (2015). Mesoporous thin films, zwitterionic monomers, and iniferter-initiated polymerization: Polymerization in a confined space. Chem. Mat..

[201] Muñoz-Santiburcio D., Marx D. (2017). Chemistry in nanoconfined water. Chem. Sci..

[202] Muñoz-Santiburcio D., Marx D. (2017). Nanoconfinement in Slit Pores Enhances Water Self-Dissociation. Phys. Rev. Lett..

[203] Huang X.L. (2018). Hydrolysis of phosphate esters catalyzed by inorganic iron oxide nanoparticles acting as biocatalysts. Astrobiology.

[204] Huang X.L. (2009). Iron Oxide Nanoparticles: An Inorganic Phosphatase. Nanocatalysts.

[205] Duval S., Branscomb E., Trolard F., Bourrié G., Grauby O., Heresanu V., Schoepp-Cothenet B., Zuchan K., Russell M.J., Nitschke W. (2020). On the why’s and how’s of clay minerals’ importance in life’s emergence. Appl. Clay Sci..

[206] Duval S., Zuchan K., Baymann F., Schoepp-Cothenet B., Branscomb E., Russell M.J., Nitschke W., Kroneck P., Sosa Torres M.E. (2021). Minerals and the emergence of life. Metals in Life Sciences.

[207] Brilmayer R., Kübelbeck S., Khalil A., Brodrecht M., Kunz U., Kleebe H.J., Buntkowsky G., Baier G., Andrieu-Brunsen A. (2020). Influence of nanoconfinement on the pka of polyelectrolyte functionalized silica mesopores. Adv. Mat. Interf..

[208] Hooks M.R., Webster P., Weber J.M., Perl S., Barge L.M. (2020). Effects of Amino Acids on Iron-Silicate Chemical Garden Precipitation. Langmuir.

[209] Ochs M., Khalil A., Frömling T., Andrieu-Brunsen A. (2021). Influence of Wettability on the Impedance of Ion Transport Through Mesoporous Silica Films. Adv. Mat. Interf..

[210] Cox B., Ness F., Tuite M. (2003). Analysis of the generation and segregation of propagons: Entities that propagate the [PSI+] prion in yeast. Genetics.

[211] Chernoff Y.O. (2004). Amyloidogenic domains, prions and structural inheritance: Rudiments of early life or recent acquisition?. Curr. Opin. Chem. Biol..

[212] Dixson J.D., Azad R.K. (2020). Prions: Roles in development and adaptive evolution. J. Mol. Evol..

[213] Glover J.R., Kowal A.S., Schirmer E.C., Patino M.M., Liu J.J., Lindquist S. (1997). Self-seeded fibers formed by Sup35, the protein determinant of [PSI+], a heritable prion-like factor of S. cerevisiae. Cell.

[214] Lupi O., Dadalti P., Cruz E., Sanberg P.R. (2006). Are prions related to the emergence of early life?. Med. Hyp..

[215] Maury C.P.J. (2009). Self-propagating β-sheet polypeptide structures as prebiotic informational molecular entities: The amyloid world. Orig. Life Evol. Biosph..

[216] Maury C.P.J. (2018). Amyloid and the origin of life: Self-replicating catalytic amyloids as prebiotic informational and protometabolic entities. Cell. Mol. Life Sci..

[217] Jackson J.B. (2016). Natural pH gradients in hydrothermal alkali vents were unlikely to have played a role in the origin of life. J. Mol. Evol..

[218] Jackson J.B. (2017). Ancient living organisms escaping from, or imprisoned in, the vents?. Life.

[219] Lane N. (2017). Proton gradients at the origin of life. BioEssays.

[220] Ross D.S. (2018). It is neither Frankenstein nor a submarine alkaline vent, it is just the Second Law. Bioessays.

[221] Branscomb E., Russell M.J. (2018). Why the Submarine Alkaline Vent is the Most Reasonable Explanation for the Emergence of Life. BioEssays.

[222] Wächtershäuser G. (2016). In praise of error. J. Mol. Evol..

[223] Herschy B., Whicher A., Camprubi E., Watson C., Dartnell L., Ward J., Evans J.R., Lane N. (2014). An origin-of life reactor to simulate alkaline hydrothermal vents. J. Mol. Evol..

[224] Sojo V., Ohno A., McGlynn S.E., Yamada Y., Nakamura R. (2019). Microfluidic reactors for carbon fixation under ambient-pressure alkaline-hydrothermal-vent conditions. Life.

[225] Hudson R., de Graaf R., Strandoo Rodin M., Ohno A., Lane N., McGlynn S.E., Yamada Y.M.A., Nakamura R., Barge L.M., Braun D. (2020). CO_2_ reduction driven by a pH gradient. Proc. Natl. Acad. Sci. USA.

[226] Russell M.J., Nitschke W., Branscomb E. (2013). The inevitable journey to being. Philos. Trans. R. Soc. Lond. B.

[227] Hansen H.C.B., Borggaard O.K., Sørensen, J. (1994). Evaluation of the free energy of formation of Fe(II)-Fe(III) hydroxide-sulphate (green rust) and its reduction of nitrite. Geochim. Cosmochim. Acta.

[228] Etique M., Zegeye A., Grégoire B., Carteret C., Ruby C. (2014). Nitrate reduction by mixed iron (II-III) hydroxycarbonate green rust in the presence of phosphate anions: The key parameters influencing the ammonium selectivity. Water Res..

[229] Constantino V.R., Pinnavaia T.J. (1995). Basic properties of Mg^2+^_1-x_Al^3+^_x_ layered double hydroxides intercalated by carbonate, hydroxide, chloride, and sulfate anions. Inorg. Chem..

[230] Hansen H.C.B., Koch C.B., Nancke-Krogh H., Borggaard O.K., Sørensen J. (1996). Abiotic nitrate reduction to ammonium: Key role of green rust. Environ. Sci. Technol..

[231] Barge L.M., Flores E., Baum M.M., VanderVelde D.G., Russell M.J. (2019). Redox and pH gradients drive amino acid synthesis in iron oxyhydroxide mineral systems. Proc. Natl. Acad. Sci. USA.

[232] Barge L.M., Jones J.P., Pagano J.J., Martinez E., Bescup J. (2020). Three-dimensional analysis of a simulated prebiotic hydrothermal chimney. ACS Earth Space Chem..

[233] Andrault D., Bolfan-Casanova N., Bouhifd M.A., Boujibar A., Garbarino G., Manthilake G., Mezouar M., Monteux J., Parisiades P., Pesce G. (2017). Toward a coherent model for the melting behaviour of the deep Earth’s mantle. Phys. Earth planet. Inter..

[234] Armstrong K., Frost D.J., McCammon C.A., Rubie D.C., Ballaran T.B. (2019). Deep magma ocean formation set the oxidation state of Earth’s mantle. Science.

[235] Agrusta R., Morison A., Agrusta R., Labrosse S., Deguen R., Alboussiére T., Tackley P.J., Dubuffet F. (2020). Mantle convection interacting with magma oceans. Geophys. J. Internatl..

[236] Mills G.C., Kenyon D. (1996). The RNA world: A critique. Orig. Des..

[237] Kurland C.G. (2010). The RNA dreamtime: Modern cells feature proteins that might have supported a prebiotic polypeptide world but nothing indicates that RNA world ever was. Bioessays.

[238] Maden B.E.H. (1995). No soup for starters? Autotrophy and the origins of metabolism. Trends Biochem. Sci..

[239] Wächtershäuser G. (1988). Before enzymes and templates: Theory of surface metabolism. Microbiol. Rev..

[240] Wächtershäuser G. (1990). Evolution of the first metabolic cycles. Proc. Natl. Acad. Sci. USA.

[241] Koonin E.V. (2007). The cosmological model of eternal inflation and the transition from chance to biological evolution in the history of life. Biol. Direct.

[242] Sharov A. (2016). Coenzyme world model of the origin of life. Biosystems.

[243] Milner-White E.J. (2019). Protein three-dimensional structures at the origin of life. Interface Focus.

[244] Lane N., Allen J.F., Martin W. (2010). How did LUCA make a living? Chemiosmosis in the origin of life. BioEssays.

[245] Jordan S., Rammu H., Zheludev I.N., Hartley A.M., Maréchal A., Lane N. (2019). Promotion of protocell self-assembly from mixed amphiphiles at the origin of life. Nat. Ecol. Evol..

[246] Kandler O., König H. (1998). Cell wall polymers in Archaea (Archaebacteria). Cell. Mol. Life Sci..

[247] Martin W., Russell M.J. (2003). On the origins of cells: A hypothesis for the evolutionary transitions from abiotic geochemistry to chemoautotrophic prokaryotes, and from prokaryotes to nucleated cells. Philos. Trans. R. Soc. Lond. B.

[248] Martin W., Russell M.J. (2007). On the origin of biochemistry at an alkaline hydrothermal vent. Philos. Trans. R. Soc. Lond. B.

[249] Sojo V., Pomiankowski A., Lane N. (2014). A bioenergetic basis for membrane divergence in archaea and bacteria. PLoS Biol..

[250] Wilson E.O. (1978). On Human Nature.

[251] Russell M.J., Zachrisson E. (1988). Chimneys, chemical gardens and feldspar horizons+pyrrhotine in some SEDEX deposits: Aspects of alkaline environments of deposition. Proceedings of the Seventh IAGOD Symposium.

[252] Filtness M.J., Butler I.B., Rickard D. (2003). The origin of life: The properties of iron sulphide membranes. Trans. Inst. Min. Metall. Sect. B.

[253] Rickard D., Luther G.W. (2007). Chemistry of Iron Sulfides. Chem. Rev..

[254] Wang Q., Barge L.M., Steinbock O. (2019). Production of Pyrophosphate Catalyzed by Mineral Membranes with Steep pH Gradients. Chemistry.

[255] Nitschke W., Russell M.J. (2010). Just Like the Universe the Emergence of Life had High Enthalpy and Low Entropy Beginnings. J. Cosmol..

[256] Wächtershäuser G. (1988). Pyrite formation, the first energy source for life: A hypothesis. Syst. Appl. Microbiol..

[257] Sleep N.H., Zahnle K., Neuhoff P.S. (2001). Initiation of clement surface conditions on the early Earth. Proc. Natl. Acad. Sci. USA.

[258] Benner S.A., Kim H.J. (2015). The case for a Martian origin for Earth life. Instruments, Methods, and Missions for Astrobiology XVII.

[259] Davies P. (1998). Did Earthlife Come from Mars. Exobiology: Matter, Energy, and Information in the Origin and Evolution of Life in the Universe.

[260] Kirschvink J.L., Weiss B.P. (2002). Mars, panspermia, and the origin of life: Where did it all begin. Palaeontol. Electron..

[261] Barras C. (2014). Water of death: Why the first life was anything but wet. New Sci..

[262] Von Helmholtz H., Kahl R. (1871). The Origin of the Planetary System. Selected Writings of Hermann von Helmholtz.

[263] Marx K. Letter to Pyotr Lavrov. In *Marx & Engels by Date—Marxists Internet Archive*, 1870–1895, Letter ID:73plv45. https://www.correspondence.ie/index.php?letters_function=4&letters_search_term=73plv45.

[264] Russell M.J. (2019). Prospecting for Life. Interface Focus.

[265] Russell M.J. (1996). Discussion of Shock, E.L. Hydrothermal systems as environments for the emergence of life. Evolution of Hydrothermal Ecosystems on Earth (and Mars).

[266] Mitchell P. (1976). Vectorial chemistry and the molecular mechanics of chemiosmotic coupling: Power transmission by proticity. Biochem. Soc. Trans..

[267] Nitschke W., Russell M.J. (2013). Beating the acetyl coenzyme A-pathway to the origin of life. Philos. Trans. R. Soc. Lond. B Biol. Sci..

[268] Russell M.J. (1968). Structural controls of base-metal mineralization in Ireland in relation to continental drift. Trans. Inst. Min. Metall. (Appl. Earth Sci. Sect. B).

[269] Russell M.J. (1972). The geological environment of post-Caledonian base-metal mineralization in Ireland. Ph.D. Thesis.

[270] Russell M.J., Tarling D.H., Runcorn S.K. (1973). Base-metal mineralization in Ireland and Scotland and the formation of Rockall Trough. Implications of Continental Drift to the Earth Sciences.

[271] Russell M.J. (1978). Downward-excavating hydrothermal cells and Irish-type ore deposits: Importance of an underlying thick Caledonian Prism. Trans. Inst. Min. Metall. (Appl. Earth Sci. Sect. B).

[272] Russell M.J. (1983). Major sediment-hosted zinc + lead deposits: Formation from hydrothermal convection cells that deepen during crustal extension. Short Course in Sediment-Hosted Stratiform Lead-Zinc Deposits.

[273] Russell M.J., Solomon M., Walshe J.L. (1981). The genesis of sediment-hosted, exhalative zinc + lead deposits. Miner. Depos..

[274] Hays S.J., Hall J., Simmons G., Russell M.J. (1988). Sealed microcracks in the Lewisian of NW Scotland: A record of 2 billion years of fluid circulation. Geol. Soc. Lond..

[275] Mills H., Halliday A.N., Ashton J.H., Anderson I.K., Russell M.J. (1987). Origin of a giant orebody at Navan, Ireland. Nature.

[276] Fallick A.E., Ashton J.H., Boyce A.J., Ellam R.M., Russell M.J. (2001). Bacteria were responsible for the magnitude of the world-class hydrothermal base-metal orebody at Navan, Ireland. Econ. Geol..

[277] Russell M.J. (1974). Manganese halo surrounding the Tynagh ore deposit, Ireland: A preliminary note. Trans. Inst. Min. Metall. (Appl. Earth Sci. Sect. B).

[278] Russell M.J. (1975). Lithogeochemical environment of the Tynagh base-metal deposit, Ireland, and its bearing on ore deposition. Trans. Inst. Min. Metall. (Appl. Earth Sci. Sect. B).

[279] Larter R.C.L., Boyce A.J., Russell M.J. (1981). Hydrothermal pyrite chimneys from the Ballynoe Baryte deposit, Silvermines, County Tipperary, Ireland. Miner. Depos..

[280] Boyce A.J., Coleman M.L., Russell M.J. (1983). Formation of fossil hydrothermal chimneys and mounds from Silvermines, Ireland. Nature.

[281] Banks D.A. (1985). A fossil hydrothermal worm assemblage from the Tynagh lead–zinc deposit in Ireland. Nature.

[282] Banks D.A., Russell M.J. (1992). Fluid mixing during ore deposition at the Tynagh base-metal deposit, Ireland. Eur. J. Miner..

[283] Boyce A.J., Fallick A.E., Fletcher T.J., Russell M.J., Ashton J. (1994). Detailed sulphur isotope studies of Lower Palaeozoic-hosted pyrite below the giant Navan Zn+Pb mine, Ireland: Evidence of mass transport of crustal S to a sediment-hosted deposit. Miner. Mag..

[284] Windman T., Zolotova N., Schwandner F., Shock E.L. (2007). Formate as an energy source for microbial metabolism in chemo-synthetic zones of hydrothermal ecosystems. Astrobiology.

[285] Etiope G., Schoell M., Hosgörmez H. (2011). Abiotic methane flux from the Chimaera seep and Tekirova ophiolites (Turkey): Understanding gas exhalation from low temperature serpentinization and implications for Mars. Earth Planet. Sci. Lett..

[286] Ranjan S., Todd Z.R., Rimmer P.B., Sasselov D.D., Babbin A.R. (2019). Nitrogen oxide concentrations in natural waters on early Earth. Geochem. Geophys. Geosyst..

[287] Airapetian V.S., Buzulukova N. (2018). Extreme space weather in time: Effects on earth. Extreme Events in Geospace.

[288] Trolard F., Bourrié G., Valaskova M., Martynkova G.S. (2012). Fougerite a natural layered double hydroxide in gley soil: Habitus, structure, and some properties. Clay Minerals in Nature: Their Characterization, Modification and Application.

[289] Russell M.J., Hall A.J., Cairns-Smith A.G., Braterman P.S. (1988). Submarine hot springs and the origin of life. Nature.

[290] Russell M.J., Daniel R.M., Hall A.J., Sherringham J. (1994). A hydrothermally precipitated catalytic iron sulphide membrane as a first step toward life. J. Mol. Evol..

[291] Russell M.J., Daia D.E., Hall A.J., Wiegel J., Adams M.W. (1998). The emergence of life from FeS bubbles at alkaline hot springs in an acid ocean. Thermophiles: The Keys to Molecular Evolution and the Origin of Life?.

[292] Zedef V., Russell M.J., Hall A.J., Fallick A.E. (2000). Genesis of Vein-Stockwork and Sedimentary Magnesite and Hydromagnesite Deposits in the Ultramafic Terranes of Southwestern Turkey: A Stable Isotope Study. Econ. Geol..

[293] Ludwig K.A., Shen C.C., Kelley D.S., Cheng H., Edwards R.L. (2011). U–Th systematics and ^230^Th ages of carbonate chimneys at the Lost City Hydrothermal Field. Geochim. Cosmochim. Acta.

[294] Russell M.J., Hall A.J., Rahman L., Turner D.M. (2001). Abiotic organic syntheses in deep submarine, alkaline hydrothermal systems catalysed by Fe^0^, mackinawite, violarite and green rust. Eleventh Annual VM Goldschmidt Conference Abstract #LPI Contribution No. 1088.

[295] Weber J.M., Barge L.M. (2021). Iron-Silicate Chemical Garden Morphology and Silicate Reactivity with Alpha-Keto Acids. ChemSystemsChem.

[296] Branscomb E., Russell M.J. (2019). On the beneficent thickness of water. Interface Focus.

[297] Astumian R.D. (2007). Design principles for Brownian molecular machines: How to swim in molasses and walk in a hurricane. Phys. Chem. Chem. Phys..

[298] Astumian R.D. (2007). Coupled transport at the nanoscale: The unreasonable effectiveness of equilibrium theory. Proc. Natl. Acad. Sci. USA.

[299] Arya S., Mukhopadhyay S. (2014). Ordered water within the collapsed globules of an amyloidogenic intrinsically disordered protein. J. Phys. Chem. B.

[300] Arya S., Singh A.K., Khan T., Bhattacharya M., Datta A., Mukhopadhyay S. (2016). Water rearrangements upon disorder-to-order amyloid transition. J. Phys. Chem. Lett..

[301] Remsing R.C., McKendry I.G., Strongin D.R., Klein M.L., Zdilla M.J. (2015). Frustrated solvation structures can enhance electron transfer rates. J. Phys. Chem. Lett..

[302] Bhullar R.K., Zdilla M.J., Klein M.L., Remsing R. (2021). Effect of water frustration on water oxidation catalysis in the nanoconfined interlayers of layered manganese oxides birnessite and buserite. J. Mat. Chem. A.

[303] Milner-White E.J., Russell M.J. (2005). Nests as sites for phosphates and iron-sulfur thiolates in the first membranes: 3 to 6 residue anion-binding motifs (nests). Orig. Life Evol. Biosph..

[304] Dalal  V., Arya S., Mukhopadhyay S. (2016). Confined Water in Amyloid-Competent Oligomers of the Prion Protein. ChemPhysChem.

[305] Camino J.D., Gracia P., Cremades N. (2020). The role of water in the primary nucleation of protein amyloid aggregation. Biophys. Chem..

[306] Russell M.J., Nitschke W. (2017). Methane: Fuel or exhaust at the emergence of life?. Astrobiology.

[307] Bols M.L., Hallaert S.D., Snyder B.E., Devos J., Plessers D., Rhoda H.M., Dusselier M., Schoonheydt R.A., Pierloot K., Solomon E.I. (2018). Spectroscopic identification of the α-Fe/α-O active site in Fe-CHA zeolite for the low-temperature activation of the methane C–H bond. J. Am. Chem. Soc..

[308] Elitzur A.C. (1994). Let there be life: Thermodynamic reflections on biogenesis and evolution. J. Theor. Biol..

[309] Qiao L., Duan G., Zhang S., Ren Y., Sun Y., Tang Y., Wan P., Pang R., Chen Y., Russell A.G. (2020). Electrochemical ammonia synthesis catalyzed with a CoFe layered double hydroxide–A new initiative in clean fuel synthesis. J. Clean. Prod..

[310] Schoepp-Cothenet B., van Lis R., Atteia A., Baymann F., Capowiez L., Ducluzeau A.-L., Duval S., ten Brink F., Russell M.J., Nitschke W. (2013). On the universal core of bioenergetics. Biochim. Biophys. Acta Bioenerg..

[311] Erastova V., Degiacomi M.T., Fraser D.G., Greenwell H.C. (2017). Mineral surface chemistry control for origin of prebiotic peptides. Nat. Commun..

[312] Cardenas M.B., Rodolfo R.S., Lapus M.R., Cabria H.B., Fullon J., Gojunco G.R., Breecker D.O., Cantarero D.M., Evaristo J., Siringan F.P. (2020). Submarine groundwater and vent discharge in a volcanic area associated with coastal acidification. Geophys. Res. Lett..

[313] Feng Y., Ovalle M., Seale J.S., Lee C.K., Kim D.J., Astumian R.D., Stoddart J.F. (2021). Molecular Pumps and Motors. J. Am. Chem. Soc..

[314] Astumian R.D., Mukherjee S., Warshel A. (2016). The physics and physical chemistry of molecular machines. ChemPhysChem.

[315] Kellosalo J., Kajander T., Kogan K., Pokharel K., Goldman A. (2012). The structure and catalytic cycle of a sodium-pumping pyrophosphatase. Science.

[316] Baykov A.A. (2020). Energy Coupling in Cation-Pumping Pyrophosphatase—Back to Mitchell. Front. Plant Sci..

[317] Holmes A.O., Kalli A.C., Goldman A. (2019). The function of membrane integral pyrophosphatases from whole organism to single molecule. Front. Mol. Biosci..

[318] Astumian R.D. (2018). Stochastically pumped adaptation and directional motion of molecular machines. Proc. Natl Acad. Sci. USA.

[319] Carter C.W. (2020). Escapement mechanisms: Efficient free energy transduction by reciprocally-coupled gating. Proteins.

[320] Carter C.W., Wills P.R. (2021). Reciprocally-Coupled Gating: Strange Loops in Bioenergetics, Genetics, and Catalysis. Biomolecules.

[321] Oster G. (2002). Darwin’s motors. Nature.

[322] Hoffmann P.M. (2012). Life’s Ratchets.

[323] Branscomb E., Russell M.J. (2013). Turnstiles and bifurcators: The disequilibrium converting engines that put metabolism on the road. Biochim. Biophys. Acta.

[324] Wander M.C., Rosso K.M., Schoonen M.A. (2007). Structure and charge hopping dynamics in green rust. J. Phys. Chem. C.

[325] Taglialegna A., Lasa I., Valle J. (2016). Amyloid structures as biofilm matrix scaffolds. J. Bacteriol..

[326] Pfammatter M., Andreasen M., Meisl G., Taylor C.G., Adamcik J., Bolisetty S., Sánchez-Ferrer A., Klenerman D., Dobson C.M., Mezzenga R. (2017). Absolute quantification of amyloid propagons by digital microfluidics. Analyt. Chem..

[327] Wang Q., Steinbock O. (2020). Materials synthesis and catalysis in microfluidic devices: Prebiotic chemistry in mineral membranes. ChemCatChem.

[328] DelloStritto M.J., Thenuwara A.C., Klein M.L., Strongin D.R. (2019). Effect of Interlayer Co^2+^ on Structure and Charge Transfer in NiFe Layered Double Hydroxides. J. Phys. Chem. C.

[329] Ding Y., Cartwright J.H., Cardoso S.S. (2019). Intrinsic concentration cycles and high ion fluxes in self-assembled precipitate membranes. Interface Focus.

[330] de Herrera A.G., Markert T., Trixler F. (2021). Abiotic nanofluidic environments induce prebiotic condensation in water. Res. Sq..

[331] Kulkarni M.B., Goel S.G. (2020). Microfluidic devices for synthesizing nanomaterials–A review. Nano Express.

[332] Möller F.M., Kriegel F., Kieß M., Sojo V., Braun D. (2017). Steep pH gradients and directed colloid transport in a microfluidic alkaline hydrothermal pore. Angew. Chem. Int. Ed. Engl..

[333] Semenov S., Semenov S.N., Kraft L.J., Ainla A., Zhao M., SBaghbanzadeh M., Campbell V.E., Kang K., Fox J.M., Whitesides G.M. (2016). Autocatalytic, bistable, oscillatory networks of biologically relevant organic reactions. Nature.

[334] Bose S.K., Lawrence C.P., Liu Z., Makarenko K.S., van Damme R.M., Broersma H.J., van der Wiel W.G. (2015). Evolution of a designless nanoparticle network into reconfigurable Boolean logic. Nat. Nanotech..

[335] Kotopoulou E., Lopez-Haro M., Calvino Gamez J.J., García-Ruiz J.M. (2021). Nanoscale anatomy of iron-silica self-organized membranes: Implications for prebiotic chemistry. Angew. Chem. Int. Ed. Engl..

[336] Roldan A., Hollingsworth N., Roffey A., Islam H.U., Goodall J.B., Catlow C.R., Darr J.A., Bras W., Sankar G., Holt K.B. (2015). Bio-inspired CO_2_ conversion by iron sulfide catalysts under sustainable conditions. Chem. Commun..

[337] Santos-Carballal D., Roldan A., De Leeuw N.H. (2020). CO_2_ reduction to acetic acid on the greigite Fe_3_S_4_{111} surface. Faraday Discuss..

[338] Katz J.E., Zhang X., Attenkofer K., Chapman K.W., Frandsen C., Zarzycki P., Rosso K.M., Falcone R.W., Waychunas G.A., Gilbert B. (2012). Electron small polarons and their mobility in iron (oxyhydr) oxide nanoparticles. Science.

[339] Chin K., Pasalic J., Hermis N., Barge L.M. (2020). Chemical Gardens as Electrochemical Systems: In Situ Characterization of Simulated Prebiotic Hydrothermal Vents by Impedance Spectroscopy. ChemPlusChem..

[340] Barge L.M., Flores E., VanderVelde D.G., Weber J.M., Baum M.M., Castonguay A. (2020). Effects of Geochemical and Environmental Parameters on Abiotic Organic Chemistry Driven by Iron Hydroxide Minerals. J. Geophys. Res. Planets.

[341] Einsle O., Messerschmidt A., Huber R., Kroneck P.M., Neese F. (2002). Mechanism of the six-electron reduction of nitrite to ammonia by cytochrome c nitrite reductase. J. Am. Chem. Soc..

[342] Arrhenius G.O. (2003). Crystals and life. Helv. Chim. Acta.

[343] Greenwell H.C., Coveney P.V. (2006). Layered double hydroxide minerals as possible prebiotic information storage and transfer compounds. Orig. Life Evol. Biosph..

[344] Bartlett S.J., Beckett P. (2019). Probing complexity: Thermodynamics and computational mechanics approaches to origins studies. Interface Focus.

[345] Pask G. (1958). Physical analogues to the growth of a concept. Mechanization of Thought Processes. Proceedings of the Symposium 10, National Physical Laboratory.

[346] Stoica A., Zebulum R.S., Keymeulen D. Mixtrinsic evolution. Proceedings of the Third International Conference on Evolvable Systems: From Biology to Hardware (ICES2000).

[347] Miller J.F., Downing K., Tufte G. (2002). Evolution in materio: Looking beyond the silicon box. Proc. NASA/DoD Evolvable Hardware Workshop.

[348] Harding S.L., Miller J.F., Rietman E.A. (2006). Evolution in materio: Exploiting the physics of materials for computation. arXiv.

[349] Stepney S. (2008). The neglected pillar of material computation. Phys. D.

[350] Stepney S. (2012). Programming unconventional computers: Dynamics, development, self-reference. Entropy.

[351] Horsman C., Stepney S., Wagner R.C., Kendon V. (2014). When does a physical system compute?. Proc. R. Soc. A.

[352] Jensen J.H., Folven E., Tufte G. (2018). Computation in artificial spin ice. Artificial Life Conference Proceedings.

[353] Adamatzky A., Meyers R.A. (2009). Reaction-diffusion computing. Encyclopedia of Complexity and Systems Science.

[354] Yu L., Mishra I.K., Xie Y., Zhou H., Sun J., Zhou J., Ni Y., Luo D., Yu F., Yu Y. (2018). Ternary Ni_2(1-x)_Mo_2x_P nanowire arrays toward efficient and stable hydrogen evolution electrocatalysis under large-current-density. Nano Energy.

[355] Arrabito G., Pezzilli R., Prestopino G., Medaglia P.G. (2020). Layered double hydroxides in bioinspired nanotechnology. Crystals.

[356] Epstein I.R., Xu B. (2016). Reaction–diffusion processes at the nano-and microscales. Nat. Nanotech..

[357] Fracchia M., Visibile A., Ahlberg E., Vertova A., Minguzzi A., Ghigna P., Rondinini S. (2018). α-and γ-FeOOH: Stability, Reversibility, and Nature of the Active Phase under Hydrogen Evolution. ACS Appl. Energy Mat..

[358] Mann S. (2008). Life as a nanoscale phenomenon. Angew. Chem. Int. Ed. Engl..

[359] Paolella A., George C., Povia M., Zhang Y., Krahne R., Gich M., Genovese A., Falqui A., Longobardi M., Guardia P. (2011). Charge transport and electrochemical properties of colloidal greigite (Fe_3_S_4_) nanoplatelets. Chem. Mater..

[360] Sano Y., Kyono A., Yoneda Y., Isaka N., Takagi S., Yamamoto G.I. (2020). Structure changes of nanocrystalline mackinawite under hydrothermal conditions. J. Mineral. Petrol. Sci..

